# ZNF521 sustains the differentiation block in MLL-rearranged acute myeloid leukemia

**DOI:** 10.18632/oncotarget.15387

**Published:** 2017-02-16

**Authors:** Giuseppe Germano, Giulia Morello, Sanja Aveic, Marica Pinazza, Sonia Minuzzo, Chiara Frasson, Luca Persano, Paolo Bonvini, Giampietro Viola, Silvia Bresolin, Claudia Tregnago, Maddalena Paganin, Martina Pigazzi, Stefano Indraccolo, Giuseppe Basso

**Affiliations:** ^1^ Foundation Institute of Pediatric Research Città della Speranza, Padova, Italya; ^2^ Department of Surgery, Oncology and Gastroenterology, University of Padova, Italy; ^3^ Department of Woman and Child Health, University of Padova, Italy; ^4^ Immunology and Molecular Oncology Unit, Istituto Oncologico Veneto IRCCS, Padova, Italy

**Keywords:** ZNF521, acute myeloid leukemia, myeloid differentiation, transcription

## Abstract

Zinc finger protein 521 (ZNF521) is a multiple zinc finger transcription factor and a strong candidate as regulator of hematopoietic stem cell homeostasis. Recently, independent gene expression profile studies have evidenced a positive correlation between *ZNF521* mRNA overexpression and *MLL*-rearranged acute myeloid leukemia (AML), leaving open the question on the role of ZNF521 in this subtype of leukemia. In this study, we sought to analyze the effect of *ZNF521* depletion on *MLL*-rearranged AML cell lines and MLL-AF9 xenograft primary cells. Knockdown of *ZNF521* with short-hairpin RNA (shRNA) led to decreased leukemia proliferation, reduced colony formation and caused cell cycle arrest in *MLL*-rearranged AML cell lines. Importantly, we showed that loss of ZNF521 substantially caused differentiation of both MLL-rearranged cell lines and primary cells. Moreover, gene profile analysis in *ZNF521*-silenced THP-1 cells revealed a loss of *MLL-AF9*-directed leukemic signature and an increase of the differentiation program. Finally, we determined that both MLL-AF9 and MLL-ENL fusion proteins directly interacted with *ZNF521* promoter activating its transcription. In conclusion, our findings identify ZNF521 as a critical effector of MLL fusion proteins in blocking myeloid differentiation and highlight ZNF521 as a potential therapeutic target for this subtype of leukemia.

## INTRODUCTION

Acute myeloid leukemias (AMLs) are heterogeneous tumor blood diseases characterized by deregulated cell proliferation, survival and differentiation of hematopoietic/stem progenitor cells [1]. One of the most aggressive subtypes of AML is characterized by the presence of translocation involving the *mixed lineage leukemia* gene (*MLL*, or *KMT2A*). *MLL* encodes an H3K4 methyltransferase that forms multiprotein chromatin-modifying complexes required in controlling transcriptional program necessary for the development and maintenance of hematopoiesis [2, 3]. Translocations that include *MLL* count more than 60 different fusion partners, which have been identified in AML, acute lymphoid leukemia, and biphenotypic or chemotherapy-related leukemias [4]. In pediatric and adult AML, the most common translocation juxtaposes the N-terminal portion of the MLL protein to the C-terminal fragment of the AF9 fusion partner in the t(9;11)(p22;q23) generating the oncogenic MLL-AF9 fusion protein [5–7]. *MLL* translocations contribute to leukemogenesis subverting self-renewal program and block of hematopoietic differentiation [5, 8]. Transformation by MLL-AF9 induced specifically aberrant expression of several transcriptional target genes involved in stem cell self-renewal, maintenance and repression of differentiation-associated genes [5, 9–10]. Among these *MLL* targets genes, such as *HOXA9* and *MEIS1*, it is well established the crucial role played in *MLL*-induced leukemia [11, 12], however many other of the genes strongly deregulated by MLL fusion proteins remain poorly characterized.

Zinc finger protein 521 (ZNF521) is a C2H2-type zinc finger transcription factor containing an amino-terminal motif that binds to the nucleosome remodeling and histone deacetylase (NuRD) complex, which is associated with transcriptional repression and conserved among other zinc finger proteins, including Friend of GATA (FOG1 and FOG2), BCL11A, and SALL family members [13]. Initially identified for its expression restricted to human CD34+ progenitor cells [14], ZNF521 has been shown to repress erythroid differentiation by inhibiting GATA-1 activity [15], and to block B-lymphoid differentiation in primary hematopoietic progenitors by antagonizing early B-cell factor 1 (*EBF1*) [16].

Deregulated expression of *ZNF521* mRNA has been observed in medulloblastoma, lymphoblastic lymphoma and acute leukemia [17–19]. Recently, knock-in mice models for *E2A-HLF* and *E2A-PBX1* involving fusion genes in B-lineage acute lymphoblastic leukemia (B-ALL) have demonstrated that enhanced expression of *Zfp521*, the murine counterpart of human *ZNF521*, cooperates to leukemia transformation, and an upregulation of *ZNF521* was found in human B-ALL samples bearing *E2A-HLF* or *E2A-PBX1* fusion oncogenes. Therefore, an altered expression of *ZNF521* may be an important cofactor contributing to hematopoietic cell transformation. Recently, high expression of *ZNF521* has been observed in pediatric AML, particularly in those cases carrying *MLL* gene rearrangements [20, 21]; however the role of ZNF521 in *MLL*-rearranged AML is currently unknown. In this study, we examined the role of ZNF521 in *MLL*-rearranged AML cell lines and primary *ex vivo MLL-AF9*-expressing cells and showed that depletion of ZNF521 impaired AML progression by inducing myeloid differentiation.

## RESULTS

### *ZNF521* is aberrantly overexpressed in pediatric *MLL*-rearranged AML

**Figure 1:**
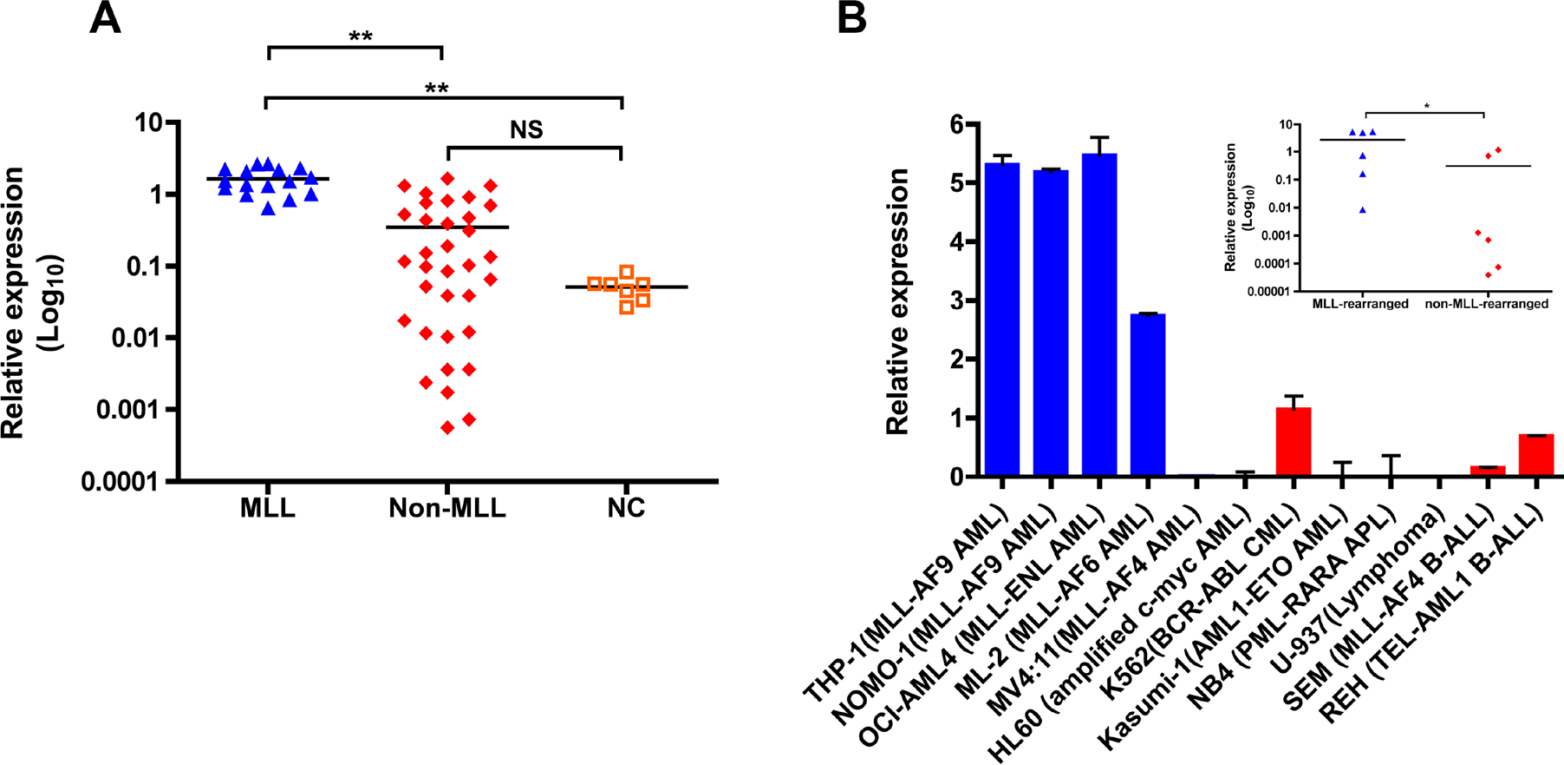
*ZNF521* is aberrantly overexpressed in *MLL*-rearranged AML (**A**) qRT-PCR for the expression of *ZNF521* in 16 *MLL*-rearranged AML (MLL), 34 non-*MLL*-rearranged AML (Non-MLL) and 7 normal control (NC). The results are normalized to *GAPDH* and analyzed by 2^−ΔCt^ method. NS, not significant, **P < 0.001, kruskal-Wallis test. (**B**) qRT-PCR analysis of *ZNF521* expression in a representative panel of 12 human leukemic cell lines normalized to *GAPDH* and analyzed by 2^−ΔCt^ method. Data are represented as mean ± SD of three independent experiments. y axis is linear. Inset, dot plots of mean *ZNF521* mRNA levels in *MLL*-rearranged and non-*MLL*-rearranged cell lines from data presented in (B). *P < 0.05, Mann–Whitney *U-test*.

Previously, by use of microarray analysis we found a frequent ZNF521 overexpression in pediatric AML with MLL rearrangements [21]. To validate these data and analyze the relationship between ZNF521 expression and distinct *MLL*-fusion genes, we performed quantitative real-time PCR (qRT-PCR) in an independent cohort of 50 pediatric AML patients (16 *MLL*-rearranged and 34 non-*MLL*-rearranged; Supplementary Table 1) and 7 normal bone marrow (BM) controls. We found that *ZNF521* was expressed at significantly higher level in AML patients with *MLL* rearrangements compared to non-rearranged AML and normal controls (*P* < 0.001, Figure 1A), The analysis of *ZNF521* expression between the most frequent *MLL* rearrangements detected in pediatric AML did not reveal significant difference based on *MLL* fusion partners (data not shown). In addition, we analyzed the expression of *ZNF521* in 6 *MLL*-rearranged and 6 non-*MLL*-rearranged human leukemic cell lines. Similarly, leukemic cell lines with *MLL* rearrangements, with the exception of those carrying *MLL-AF4* fusion transcripts, showed significantly higher *ZNF521* mRNA levels compared to cell lines with other abnormalities (*P* < 0.05, Figure 1B). Thus, our data indicate that ZNF521 is likely involved in *MLL*-mediated transformation in AML.

### *ZNF521* depletion reduces cell viability and causes cell cycle arrest without inducing apoptosis of *MLL*-rearranged AML cell lines

**Figure 2:**
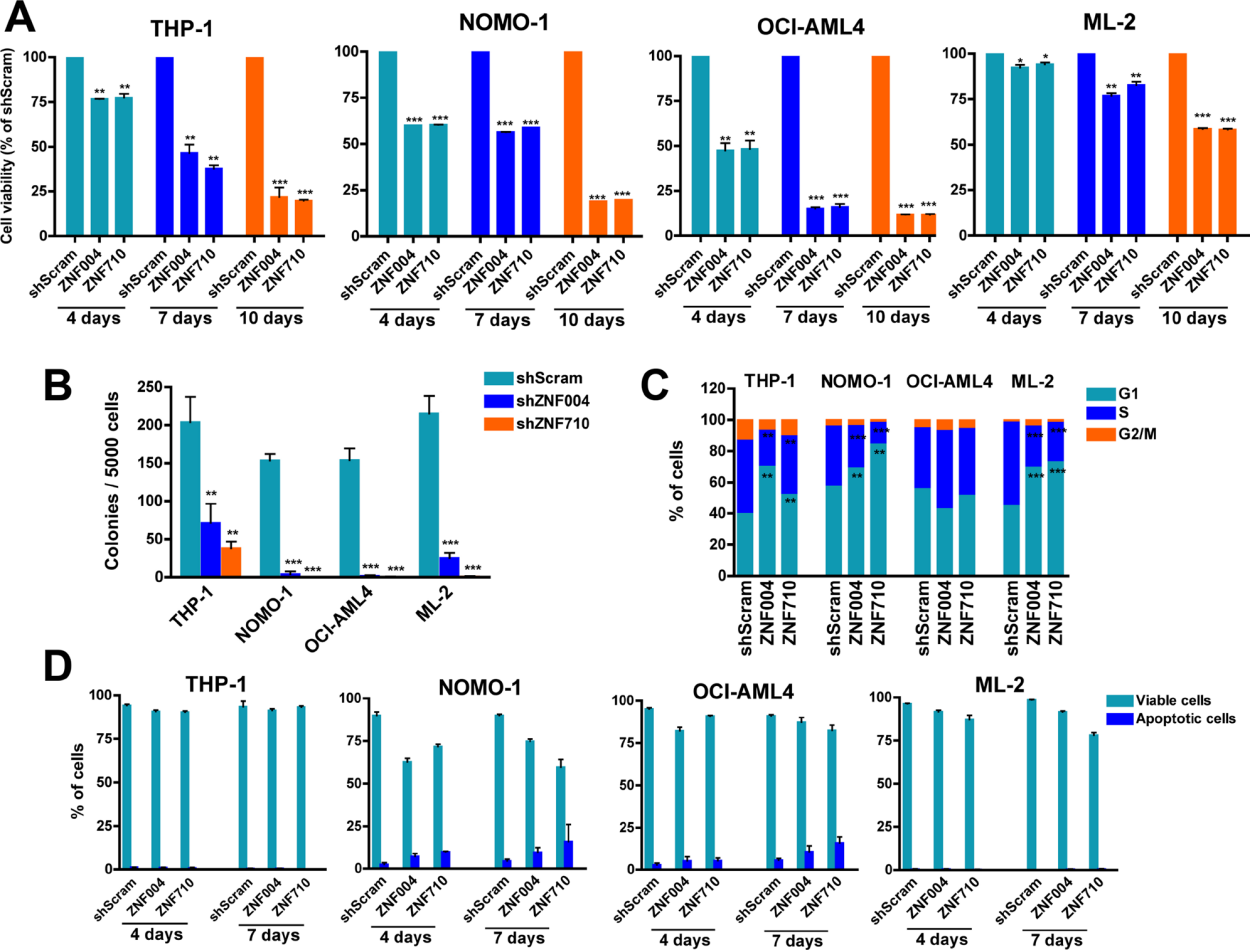
*ZNF521* depletion impairs cell proliferation, induces cell cycle arrest but not apoptosis in *MLL*-rearranged cell lines (**A**) MTT cell viability assay in the MLL leukemic cells THP-1, NOMO-1, OCI-AML4 and ML2 transduced with *ZNF521* shRNAs (ZNF004 or ZNF710) or non-targeting scramble control (shScram). GFP+ cells were sorted 4 days after transduction and placed in appropriate medium. Graphs show percentage of GFP+ cells measured at day 4, day 7 and day 10, normalized to the percentage of shScram cells. Data are represented as mean ± SD of at least three independent experiments. *P < 0.05, **P < 0.001, ***P < 0.0001, *t-test*. (**B**) Colony formation of GFP+ cells transduced with *ZNF521* shRNAs or shScram. Error bars represent mean ± S.D. of three independent experiments. **P < 0.001, ***P < 0.0001, *t-test*. (**C**) Cell cycle distribution at day 7 of *ZNF521* knockdown cells and control shScram of gated GFP+ cells. Data are represented as mean ± SD of three independent experiments. **P < 0.001, ***P < 0.0001, *t-test*. (**D**) Percentage of apoptotic cells (Annexin V+/DAPI- and Annexin V+/DAPI+) measured after 4 and 7 days post-transduction of gated GFP+ cell population. Data are represented as mean ± SD of three independent experiments.

To determine whether *ZNF521* is functionally important in *MLL*-rearranged AML, we first examined the effects of *ZNF521* knockdown on the cell proliferation using a panel of human *MLL*-rearranged AML cell lines, including, THP-1, NOMO-1 (both expressing *MLL-AF9*), ML-2 (expressing *MLL-AF6*) and OCI-AML4 (expressing *MLL-ENL*). To suppress *ZNF521*, we used GFP-tagged lentiviral vectors expressing anti-ZNF521 shRNAs (ZNF004 and ZNF710) or a non-targeting shRNA sequence (shScram). After assessing transduction efficiency by flow cytometry (range 30–80%) (Supplementary Figure 1 and data not shown), GFP-positive cells were sorted and maintained under standard cell culture conditions for subsequent analysis. As expected, in all four cell lines downregulation of *ZNF521* varied between 60% and 75% compared to *ZNF521* mRNA expression in shScram-transduced cells, and this correlated with a decrease in ZNF521 protein amount (Supplementary Figure 2). In addition, *ZNF521* knockdown progressively reduced viability of all the transduced cell lines (Figure 2A), and it inhibited colony formation ability of *MLL*-rearranged cells, measured 2 weeks after transduction (Figure 2B). In order to get a deeper insight, cell cycle analysis and apoptosis induction were assessed in GFP-positive *MLL*-rearranged cells. At day 7, we observed an accumulation of cells in G1 phase (17%–77%) in three out of four cell lines (THP-1, NOMO-1 and ML-2) expressing anti-ZNF521 shRNAs. This was most likely due to S phase reduction (from 29% to 65%) rather than G2/M alterations (Figure 2C). However, annexin V/DAPI assay measured at day 4 and day 7 demonstrated that *ZNF521* knockdown did not caused increased apoptosis (Figure 2D), suggesting that ZNF521 may be involved in proliferation and differentiation of *MLL*-rearranged cells rather than in cell survival. To substantiate this hypothesis, GFP-sorted transduced THP-1 and ML-2 cells were collected on glass slides by cytospin and stained with antibodies against p21 (CDKN1A) and p27 (CDKN1B) cell cycle inhibitors [22]. At day 7, we observed an increase of both p21 and p27 protein expression in *ZNF521* knockdown cells, suggesting a prolonged G1/S transition as the main reason for the aforementioned cell cycle arrest (Supplementary Figure 3). Taken together, these findings indicate that *ZNF521* expression is essential in the growth potential of *MLL*-rearranged AML cell lines.

### Depletion of *ZNF521* induces myeloid differentiation of *MLL*-rearranged AML cell lines

**Figure 3:**
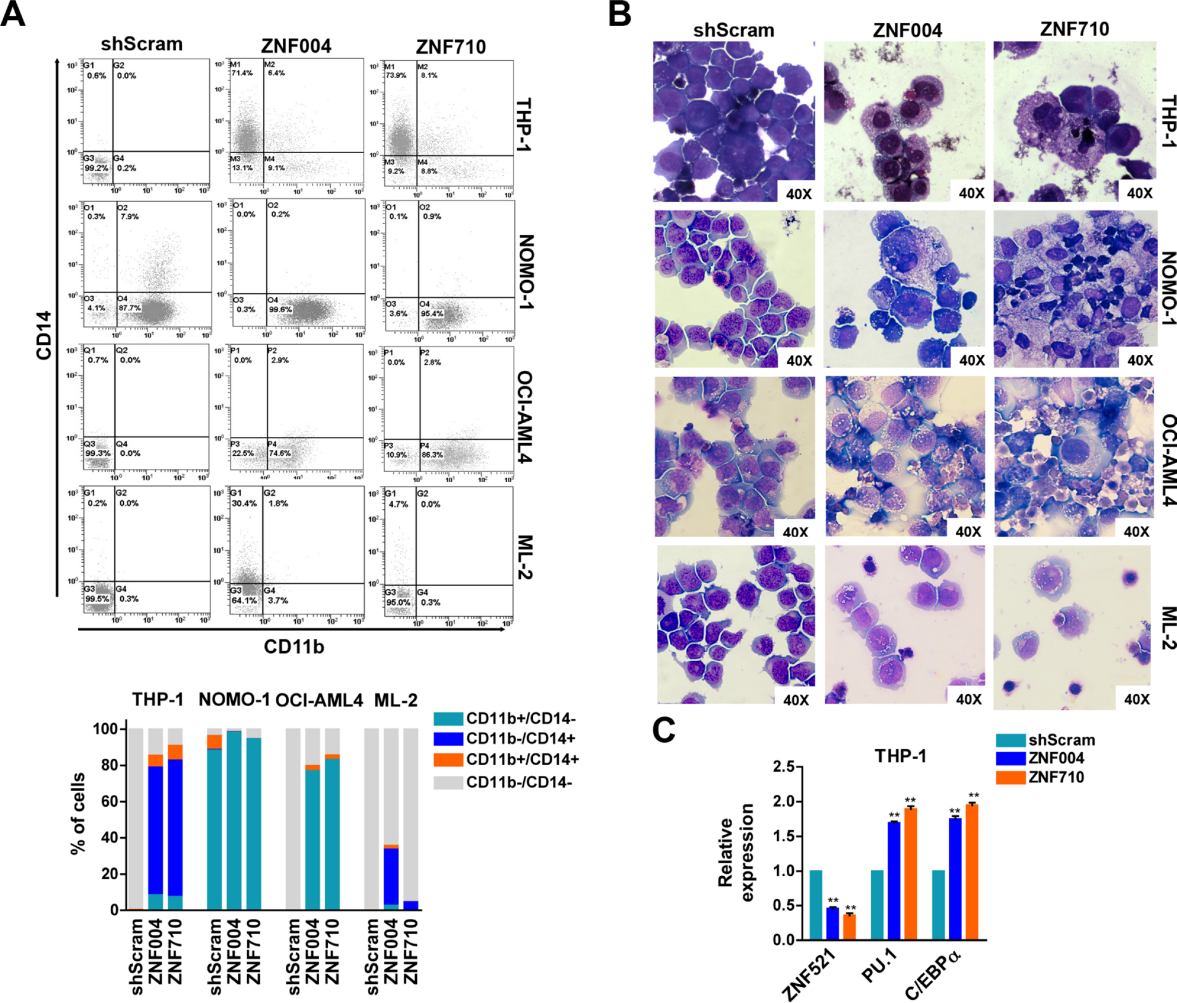
*ZNF521* depletion induces myelomonocytic differentiation in *MLL*-rearranged cell lines (**A**) Representative flow cytometry dot plots of gated GFP+ cells analyzed for CD11b and CD14 expression after 7 days of transduction. The mean percentage of CD11b+/CD14−, CD11b−/CD14+, CD11b+/CD14+ and CD11b−/CD14− cells of three biological replicates are shown below. (**B**) Representative Wright-Giemsa staining of cytospin preparations at day 7 of THP-1, NOMO-1, OCI-AML4 and ML2 GFP+ cells transduced with *ZNF521* shRNAs or shScram. Original magnification, × 40. (**C**) qRT-PCR on THP-1 GFP+ cells for the expression of *ZNF521*, *PU.1* and C/EBP*α* at day 7 post transduction with ZNF521 shRNAs or shScram. The results are relative to shScram-transduced cells, normalized to *GAPDH* and analyzed by 2^−ΔΔCt^ method. Data are represented as mean ± SD of three independent experiments. **P < 0.01, *t-test*.

Given that ZNF521 can regulate lineage progression of different cell types, including hematopoietic cells [14–16], we analyzed whether *ZNF521* depletion might influence differentiation in *MLL*-rearranged leukemic cells. Flow cytometry analysis of CD11b and CD14 myeloid markers was then performed on GFP-positive cells and revealed a change of these markers in 3 out of 4 cell lines transduced with *ZNF521* shRNAs (Figure 3A). The phenotypic changes were also sustained by a more mature macrophage-like morphology observed in all these cell lines upon *ZNF521* depletion as compared with transduced control cells (Figure 3B). Additionally, maturation induced by *ZNF521* depletion was also supported by upregulation of *C/EBPA* and *PU.1* mRNA levels, two myeloid differentiation markers (Figure 3C). Furthermore, a downregulation of *ZNF521* expression occurred in response to treatment with all-*trans* retinoid acid (ATRA) and with Securinine, two differentiation agents administered to THP-1 and NOMO-1 AML cells, respectively (Supplementary Figure 4). In particular, ATRA and Securinine, previously tested on these cell lines by others [23, 24], were able to reduce ZNF521 mRNA and protein expression, and stimulate *MLL*-rearranged cell differentiation, supporting the hypothesis that ZNF521 is required to maintain those cells in an undifferentiated state.

### Effects of *ZNF521* depletion in patient-derived AML xenograft cells

**Figure 4:**
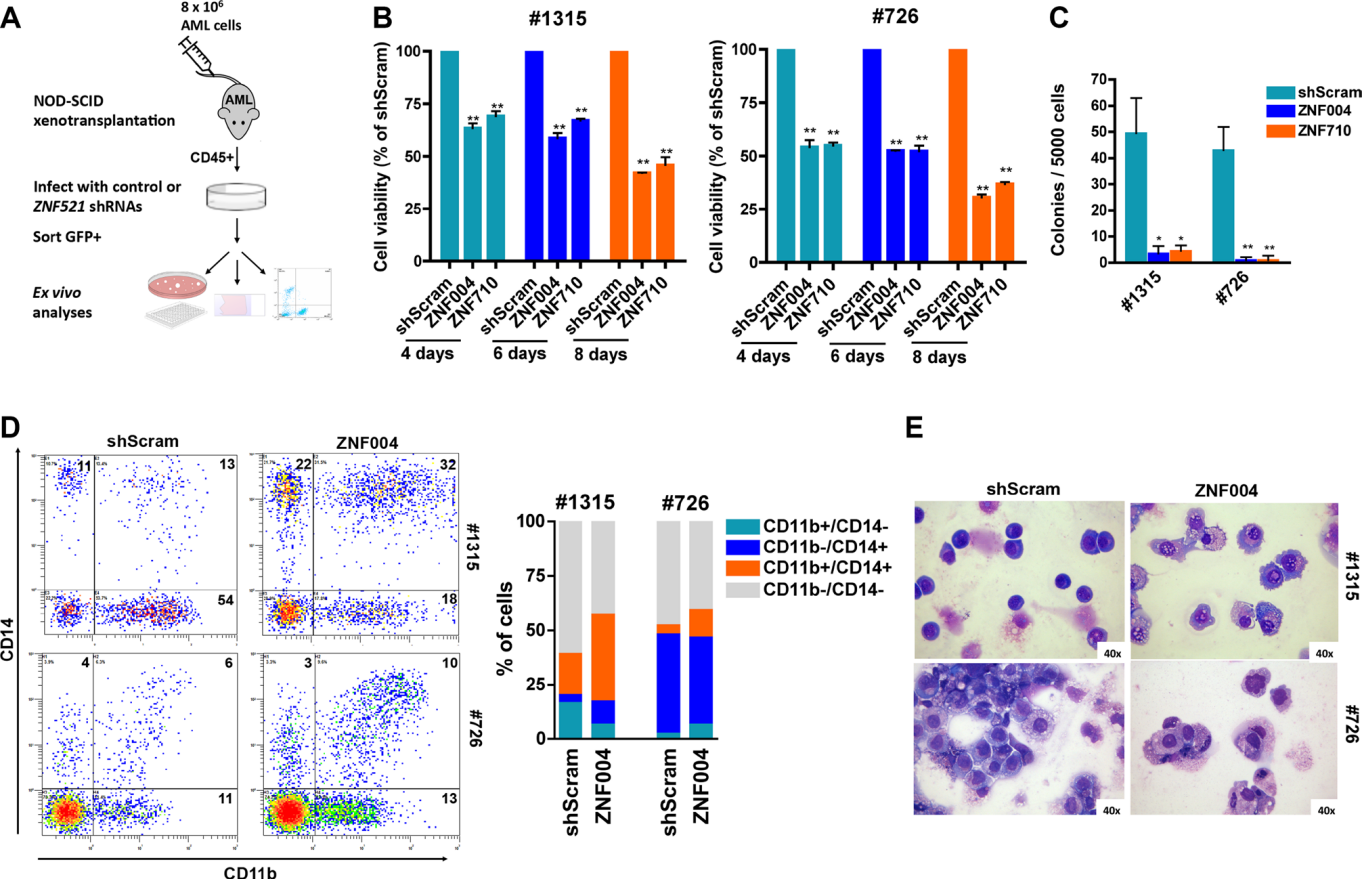
*ZNF521* depletion impairs cell growth and induces differentiation on primary MLL-AF9 AML patient-derived xenograft cells (**A**) Flow chart of experimental procedure for analyzing the role of *ZNF521* in *ex vivo* cells obtained from patient-derived xenografts. Leukemic cells from patient #726 or patient #1315 were isolated from primary AML mice and infected with lentivirus encoding an shRNA to *ZNF521* or shScram. Four days after transduction cells were FACS-sorted for GFP expression and cultured. (**B**) MTT cell viability assay in *ex vivo* cells. Data are represented as mean ± SD of three independent experiments. **P < 0.001, *t-test*. (**C**) Clonogenic growth of transduced *ex vivo* GFP+ cells following 14 days in methylcellulose culture. Data are shown as the means ± SD for triplicate analyses. *P < 0.05, **P < 0.001, *t-test*. (**D**) Representative flow cytometry dot plots showing expression of CD11b and CD14 in human CD45+ cells in the GFP+ cells population. Numbers indicate percentage of the four populations. The mean percentage of CD11b+/CD14−, CD11b−/CD14+, CD11b+/CD14+ and CD11b−/CD14− populations of three biological replicates are shown in the right panel. (**E**) Representative Wright-Giemsa-stained cytospins of *ex vivo* GFP+ cells at day 4 post transduction with ZNF521 shRNA (ZNF004) or shScram. Original magnification, × 40.

To extend our findings to primary cells containing MLL-AF9 oncogene, we transduced *ZNF521* shRNAs in *ex vivo* cells obtained from patient-derived xenografts (Figure 4A). Two out of four patients harboring *MLL-AF9* fusion protein (Supplementary Table 3) resulted in successful engraftment into NSG mice. The kinetics of such engraftment, measured by percentage of human CD45+ cells in the peripheral blood varying between 22.3% to 42.2%, ranged from 47 to 67 days and led to expansion of leukemic cells with the same immunophenotype and cytogenetic features of the original patient sample (data not shown). *Ex vivo* experiments demonstrated that *ZNF521* depletion strongly impaired viability and colony formation of mononuclear cells obtained from two primary MLL-AF9 AML patient-derived xenografts (Figure 4B, 4C). Most importantly, an increased expression of myeloid differentiation markers CD11b and CD14 (Figure 4D) and morphological features of mature monocytes/macrophage blast-like was observed (Figure 4E). These findings suggest that *ZNF521* overexpression is critical to maintain an immature phenotype consistent with the *MLL*-rearranged cell lines results.

### Gene expression changes after *ZNF521* depletion in THP-1 cells

**Figure 5:**
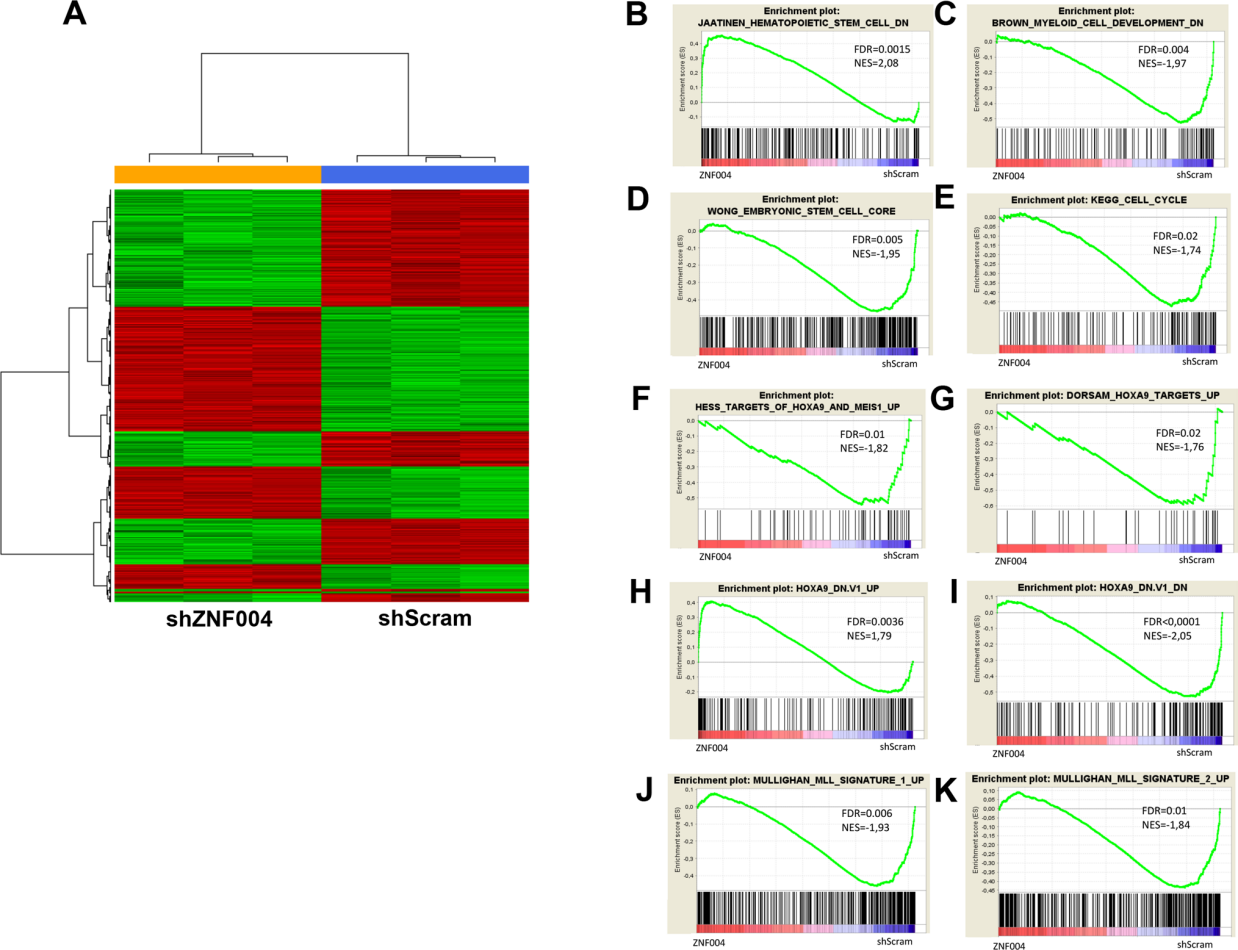
Microarray results of *ZNF521* depletion in THP-1 cells (**A**) Hierarchical clustering analysis of differently gene expression profiles associated with transduced THP-1 cells with *ZNF521* shRNA (ZNF004) or control shScram after 4 days of transduction. Each column represents a sample and each row represent a gene. Relative levels of gene expression are depicted with a color scale where red represents the highest level of expression and green represents the lowest level. (**B**–**E**) GSEA plot showing gene expression signature of (B) negative enrichment of cell cycle signature, (C) negative enrichment of downregulated genes in myeloid cell development signature, (D) positive enrichment of downregulated genes in HSCs signature and (E) negative enrichment of embryonic stem cell core signature. (**F**, **G**) GSEA plot showing negative enrichment of *MLL* signature up-regulated genes in pediatric AML. (**H–K**) GSEA showing enrichment of upregulated genes in *HOXA9* up-regulated (H) and down-regulated (I) signatures in *HOXA9* knockdown cells, (J) positive enrichment of *HOXA9* targets up-regulated and (K) negative enrichment of *HOXA9* targets down-regulated in hematopoietic stem cells. The normalization enrichment score (NES) and the false discovery rate (FDR) values are indicated in each panel. Red and blue color bars indicated the positive and negative enrichment, respectively.

To investigate the gene expression pattern in MLL-AF9 AML cells expressing high levels of *ZNF521*, we performed microarray analysis of shZNF521- or shScram-transduced THP-1 cells. Since that the differentiation was overt after 7 days of transduction as reported above, we performed gene expression profiling at day 4 after transduction. A total of 158 genes showed a significant change of expression (>1.5-fold change, FDR< 0.05), 58 were upregulated while 100 were downregulated (Figure 5A and Supplementary Table 4). Gene Set Enrichment Analysis (GSEA) confirmed that *ZNF521* depletion affected cell cycle progression and cell fate differentiation related genes [25] (Figure 5B, 5C). These results showed also positive enrichment of genes downregulated in CD133+ HSCs when compared with the CD133- cell [26], and negative enrichment of embryonic stem cells (ESC) associated genes [27] (Figure 5D, 5E). The enrichment of stemness-related genes found by our analysis is in line with proposed role of ZNF521 in the regulation of hematopoietic stem cell homeostasis [28]. Furthermore, GSEA revealed a negative enrichment with genes that are upregulated in *MLL*-rearranged pediatric AML compared with non-*MLL*-rearranged AML [29] (Figure 5F, 5G). Interestingly, the *ZNF521* depletion gene set revealed positive enrichment with genes that are upregulated in hematopoietic precursors conditionally expressing *HOXA9* and *MEIS1*, including *HOXA9* target genes upregulated in hematopoietic stem cells [8, 30] (Figure 5H, 5I). In addition, genes up-regulated or downregulated upon knockdown of *HOXA9* [11] were also similarly regulated in *ZNF521*-transduced THP-1 cells (Figure 5J, 5K). Considering individual genes, we found deregulated genes with known relevance in *MLL*-fusion-mediated AML (*TET1*, *CDK6* and *Musashi2*) [31–33] and in myeloid progenitors differentiation (CD14 and MEF2A) [34] (Supplementary Figure 5). Taken together, these results indicate that *ZNF521* expression negatively modulates genes involved in myeloid differentiation, and is required to maintain expression programs associated with *MLL*-induced transformation.

### *ZNF521* gene promoter is activates by MLL fusion proteins

**Figure 6:**
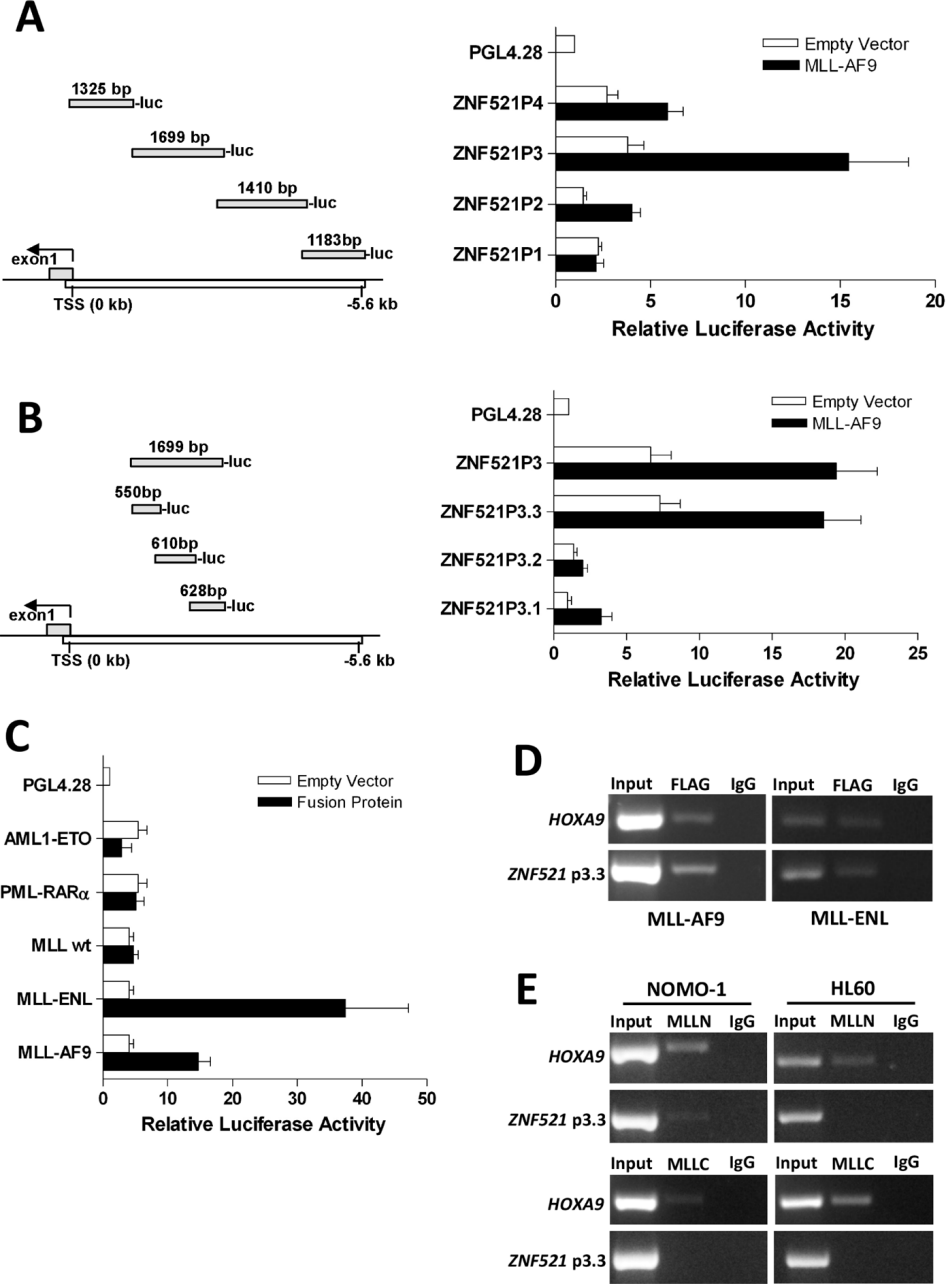
MLL-AF9 and MLL-ENL fusion oncoproteins bind to *ZNF521* promoter (**A**) An illustration of the 4 fragments representing 5058 bp of *ZNF521* promoter and their positions are indicated in the left panel. The numbers above each part are referred to the length (bp) of the genomic fragment that was PCR amplified and then cloned upstream of the luciferase coding sequence (luc) of PGL42.8 plasmid. In the right panel, horizontal bars represent the luciferase activity generate by each construct following transient transfection in 293T cells with *MLL-AF9* plasmid. (**B**) An illustration of the 3 fragments of *ZNF521P3* and their respective positions are shown in the left panel. The numbers above each part are referred to the length (bp) of the genomic fragment cloned into PGL42.8 plasmid. In the right panel, horizontal bars represent the luciferase activity generate by each construct as described in A. (**C**) Luciferase activity of the *ZNF521P3.3* fragment after transient transfection in 293T cells with AML1-ETO, PML-RAR*α*, wild-type (WT) MLL, MLL-ENL or *MLL-AF9* is shown. For each panel (A, B and C), luciferase activity is expressed relative to the empty vector of each expression plasmid (white bars) and normalizes to Firefly/Renilla luciferase activities considering the empty pGL42.8 vector as 1. Data are represented as mean ± SD of three independent experiments. (**D**) Both MLL-AF9 and MLL-ENL fusion oncogenes associates with the *ZNF521P3.3* promoter region. ChIP assays were performed with the crossed-linked genomic DNA isolated from 293T cells transfected with either *Flag-MLL-AF9* or *Flag-MLL-ENL* and using anti-Flag and anti-IgG antibodies. Normal IgG was used as a negative control. Input DNA from sonicated chromatin and immunoprecipitated DNA were subjected to PCR amplification with primers spanning the *ZNF521P3.3* promoter region. PCR amplification with primers specific to the HOXA9 promoter region was used as positive control. Data from a representative of three replicate experiments are shown. (**E**) ChIP analysis of the ZNF521P3.3 promoter in NOMO-1 cells, which express MLL-AF9 and HL60 cells, which express WT MLL but not MLL-AF9, using antibodies directed to the N-terminus of MLL (MLLN; upper panel), C-terminus of MLL (MLLC; lower panel) or IgG. Immunoprecipitated chromatin samples were analyzed by PCR using primers corresponding to promoter *ZNF521P3.3* region. Note that no PCR product for *ZNF521* promoter was obtained when anti-MLLC, which recognizes only the WT MLL but not MLL-AF9, was used for immunoprecipitation.

Finally, to investigate the molecular mechanism that upregulates *ZNF521* in *MLL*-rearranged AML, we performed luciferase reporter and ChIP assays using the Flag-tagged *MLL-AF9* expression plasmid. To this end, we generated a series of constructs in which 5.0 kb of the genomic region upstream of the *ZNF521* transcription start site (TSS) was subdivided in 4 fragments (*ZNF521P1*, *ZNF521P2*, *ZNF521P3* and *ZNF521P4*) and inserted into a pGL4-basic reporter plasmid (Figure 6A). Luciferase assays in 293T cells showed that MLL-AF9 strongly activated the promoter region that lay between -1.3 to -3 kb (ZNF521P3) of the TSS (Figure 6A). To further confirm the region of ZNF521 activated by MLL-AF9, we generated 3 constructs (*ZNF521P3.1*, *ZNF521P3.2* and *ZNF521P3.3*) spanning the *ZNF521P3* fragment (Figure 6B). We found that the *pGL4-ZNF521P3.3* construct showed the highest luciferase activity (Figure 6B), indicating that the *MLL-AF9* responsive elements likely reside between −1.0 and −1.6 kb upstream of the ZNF521 TSS. Furthermore, to determine whether *ZNF521* activation was MLL fusion-dependent, we performed *ZNF521*-driven luciferase reporter assay in another *MLL* fusion gene (*MLL-ENL*) and in two non-*MLL*-associated fusion genes such as *AML1-ETO* and *PML-RARAα*. We observed that both *AML1-ETO* and *PML-RARAα* yielded only a minimal luciferase activity compared with MLL-ENL that showed even a higher promoter binding affinity than MLL-AF9 (> 2.5 fold) (Figure 6C). Besides, wild-type (WT) MLL did not affect luciferase activity under the same settings, providing evidence that only MLL-fusion proteins likely activate *ZNF521* expression (Figure 6C). Consistent with these results, ChIP analyses showed that both MLL-AF9 and MLL-ENL bind to *ZNF521* promoter region in transfected 293T cells (Figure 6D). In order to validate MLL-AF9 binding to the *ZNF521* promoter in AML cells, we performed ChIP with lysate from NOMO-1 and HL60 cell lines that endogenously expressing MLL-AF9 and WT MLL, respectively. Since that MLL-AF9 lacks the MLL-C portion of WT MLL, an anti-MLL N-terminal (MLLN) and an anti-MLL C-terminal (MLLC) antibodies were used for this experiment. ChIP assays showed that MLLN bound specifically to the *ZNF521* promoter region in NOMO-1 but not in HL60 (Figure 6E; upper panel). By contrast, there was not apparently association with MLLC and *ZNF521* in both NOMO-1 and HL60 cells (Figure 6E; lower panel). Together, these findings demonstrated that *ZNF521* promoter is specifically bound by MLL-AF9, and provide further evidence that MLL fusion oncoproteins may drive aberrant expression of ZNF521, which may in turn lead to a block in differentiation.

## DISCUSSION

We present data showing that pediatric AML patients carrying *MLL* translocations have a significantly upregulation of *ZNF521* expression independently of the fusion partner involved in the translocation with *MLL*. The overexpression of *ZNF521* is a robust transcriptional feature of *MLL*-rearranged AML, consistent across independent adult and pediatric microarray datasets [19–21]. From these data, we started our study with the aim to decrypt the ZNF521 function as transcription factor in *MLL*-rearranged AML, and understand if it might deserve attention as potential therapeutic target.

A major hallmark of leukemia and a consequence of MLL fusion proteins expression is a block in hematopoietic differentiation [35]. On this way, our data show that the most relevant effect of *ZNF521* depletion was to enhance myeloid differentiation of leukemia cells as evidenced by changes in cell morphology, immunophenotype and increase of a myeloid-specific gene expression in *MLL*-rearranged cell lines and primary cells. The requirement of ZNF521 in the maintenance of an undifferentiated status associated with *MLL*-rearranged AML was also supported by the fact that *ZNF521* expression drastically decreased upon treatment with specific differentiation-induced agents, such as ATRA. The observed growth defect, cell cycle arrest and reduced colony formation upon *ZNF521* depletion were secondary to cells entering into a differentiation program. Thus, MLL fusion proteins might promote leukemogenesis not only by *HOXA9* and *MEIS1* upregulation, but also by keeping the *ZNF521* overexpressed, which in turn contributes to a block of differentiation or to the maintenance of an undifferentiated state of leukemia cells.

Consistent with this finding, others have reported that loss of *ZNF521* enhanced erythroid differentiation and increased B-lineage maturation in cell lines and primary hematopoietic progenitor cell, respectively [15, 16]. Moreover, it is well established that Zfp521, the mouse counterpart of human ZNF521, in other cellular contexts including, embryonic stem cells (ESCs), neural cells, osteoblasts and chondrocytes mainly function to control cell differentiation of primitive or mature cells by modulating the activity of specific transcription factors [36–39]. Consistent with these findings, our GSEAs in THP-1 cells depleted for *ZNF521* showed enrichment of hematopoietic stem cells (HSCs)- and ESC-associated downregulated genes and sets of genes associated with differentiation program [25, 26]. Based on these findings, we assumed that ZNF521 has not only a role in promoting self-renewal and maintenance of HSC but it also acts in MLL rearranged AML. Furthermore, our results support the direct activation of ZNF521 by MLL fusion proteins increasing the importance of this transcription factor in the transformation of the leukemia cells. In fact, we showed enrichment of set of genes related to MLL fusion-dependent transformation signatures as well as to HOXA9-mediated gene expression program [11, 30, 40]. Thus, the events documented after *ZNF521* depletion, which in part resemble what has been previously observed in *MLL*-rearranged cells after loss of *HOXA9*, gave a further support that ZNF521 plays a critical role in *MLL*-fusion-mediated leukemia. Interestingly, the expression of either *HOXA9*, a canonical downstream target for *MLL*-rearranged leukemia [10, 41] or *ZNF521* have been shown to be restricted in CD34+ progenitor cells [28, 30].

Nevertheless, in our gene-expression analysis, loss of *ZNF521* does not affect *HOXA9* expression, implying that both are MLL-dependent but might act in a non-mutually exclusive and additive manner.

Supporting the idea that ZNF521 is particularly required for MLL-mediated leukemia, our data of luciferase reporter and ChIP assays revealed that *ZNF521* is a direct target of both MLL-AF9 and MLL-ENL fusion proteins. We defined a genomic region of 555 bp in 5′ ZNF521 promoter that is thought to be crucial for ZNF521 activation by MLL fusion proteins. This finding is consistent with prior observations that showed how the modulation of *MLL-AF9* levels resulted in concordant changes in *ZNF521* expression in different human *in vitro* models [42, 43]. Surprisingly, the inspection of ChIP-seq data from Bernt et al. [44] did not show peak in the vicinity of the *Zfp521* gene in an MLL-AF9 mouse leukemia model. Of note, this is also observed for other well-known targets of MLL fusion proteins such as *EVI1* and *PLZF* [45, 46] About ZNF521, this can be explained by the different approaches used, and the fact that in mouse BM *Zfp521* is primarily expressed in the HSC fraction and significantly reduced in granulocyte-monocyte-progenitor cells (GMPs) (http://servers.binf.ku.dk/bloodspot/?gene=ZFP521&dataset=nl_mouse_data), in which the analysis has been done. Future ChIP-seq experiments on human transformed HSC will likely shed further light on *ZNF521-MLL-AF9* target gene specificity.

In summary, this study unravels the anti-differentiation function of ZNF521 in MLL-rearranged cells and showed the mechanism by which ZNF521 participates in MLL-fusion mediated transformation. This data also indicate that ZNF521 is highly expressed in the majority of MLL-rearranged AML pediatric patients, and thus ZNF521 could be a potential molecular target for this subtype of aggressive leukemia.

## MATERIALS AND METHODS

### Patient samples and cell lines

All of the pediatric AML patient samples were obtained at the time of diagnosis from the University-Hospital of Padua and stratified according to the AIEOP AML 2002/01 protocol AML 2002/01 [47]. Patient characteristics are listed in Supplementary Table 1. Seven BM samples from healthy donors were obtained as control. All human myeloid cell lines (THP-1, NOMO-1, OCI-AML4, ML2, HL60, K562, Kasumi-1, NB4, U-937, SEM and REH) were obtained from DSMZ (Braunschweig, Germany) and 293T cells were obtained from ATCC (Manassas, VA, USA). All cell lines were maintained under standard conditions suggested by the manufacturer.

### Quantitative real time PCR

Total RNA was extracted with Trizol reagent (Invitrogen) and reverse transcribed into cDNA using the Superscript III First-Strand Synthesis System (Life Technology). The mRNA levels of *ZNF521*, *PU.1*, *CEBPa*, *HOXA9* and *MEIS1* were measured by quantitative RT-PCR (qRT-PCR) with SYBR green on an AB 7900HT real time system (Applied Biosystem) using the comparative C_t_ method and the *GAPDH* gene expression as internal control [48]. The primer sequences for quantitative qRT-PCR are listed in Supplementary Table 2.

### Lentiviral shRNA vector, transduction and FACS-sorting

For knockdown studies, two shRNAs against *ZNF521* and a control scrambled shRNA (shScram) were used (Mission pLKO.1-puro-CMV-TurboGFP system, Cat Number TCRN0000229710 and TCRN0000229004, Sigma-Aldrich) (see Supplementary Table 2 for shRNA sequences). Lentiviral cell transduction was performed as described previously [49]. After culture in fresh medium, GFP-positive cells were sorted 96 hours after infection using a MoFlo XDP cell sorter (Beckman Coulter) and used for experiments. Alternatively, cells were gated for GFP expression and subjected to flow cytometry analyses. *ZNF521* knockdown efficiency was measured by qRT-PCR and western blot analyses.

### Plasmids constructs, transient transfection and luciferase assay

pMSCV-neo-Flag-MLL-AF9, MSCV-PML-RARA-IRES-GFP, MSCV-AML1-ETO-GFP, pCMVMLL-3xFlag and pCMVMLL-ENL-3xFlag have been previously described [42, 50–51]. Flag-tagged proteins were previously verified by Western blot with anti-Flag M2 antibody (Sigma), as well as the GFP-tagged proteins by expression of green fluorescence protein (GFP) *in vitro*. The procedure to generate the various constructs of the *ZNF521* promoter into *pGL4.28* plasmid (Promega) is described in Supplementary Materials and Methods. For luciferase assay, 293T cells were cotransfected with 0.5 μg of the reporter plasmid, 1 μg of expression plasmid or empty vector and 0.5 μg of *Renilla* luciferase reporter vector (Promega) as internal control for normalization of transfection efficiency, for a total of 2 μg of combined plasmids per well. The cells were then harvested at 48 hours after transfection using a Dual-Luciferase reporter assay system (Promega) and the Victor3 TM 1420 Multilabel Counter (PerkinElmer). Data are presented as the mean ratio for triplicate experiments.

### Chromatin immunoprecipitation (ChIP) and PCR detection

ChIP assay was performed using the Imprint Chromatin Immunoprecipitation kit (Sigma), according to the manufacturer's protocol with minor modifications. Briefly, 293T cells (3.5 × 10^6^ cells) were transfected with 10 μg of Flag-MLL-AF9 or Flag-MLL-ENL expression plasmids. 48 hours post transfection, were cross-linked with 1% formaldehyde (Sigma) for 15 minute at room temperature. Subsequently, the lysed cells were isolated and sonicated on ice to shear DNA into fragments of 200 bp to 1 kb. Then, the chromatin complexes were incubated into pre-treated Stripwells (Sigma) with anti-Flag M2 monoclonal antibody (Sigma), or normal mouse IgG (Sigma) as indicated. The input DNA was isolated from sonicated lysates before immunoprecipitation as a positive control. Purified DNA was then resuspended in TE buffer (10 mM Tris-HCL and 1 mM EDTA, pH 8.0) for PCR. ChIP assay from 2 × 10^6^ of *MLL-AF9*-expressing NOMO-1 cells or HL60 cells was performed as above reported using a N-terminal MLL monoclonal antibody (Santa Cruz Biotechnology) or a C-terminal MLL polyclonal antibody (Sigma) or a mouse IgG (Sigma) as indicated. Purified ChIP DNA was amplified by regular PCR. Primers amplifying the ZNF521 promoter region and the HOXA9 promoter region used for the ChIP PCR are listed in Supplementary Table 2.

### Microarray analysis

Total RNA from sorted THP-1 cells transduced with shRNAs was isolated using Trizol as above reported and processed for microarray analysis using the Affymetrix GeneChip 3′IVT express Kit (Affimetrix) after RNA quality control using Agilent 2100 Bioanalyzer (Agilent). Gene expression profile was performed using a Human Genome U133 2.0 Plus chip (Affymetrix), as previously described [52]. The data were RMA-normalized using R software (http://www.r-project.org/) with BioConductor package (www.bioconductor.org). Shrinkage *t* test was used to identify differentially expressed genes between shScram and shRNA ZNF521 THP-1 cells selected with a local FDR < 0.05 (FDR). Hierarchical clustering analyses were performed using Euclidian distance and Ward's methods. Gene set enrichment analysis (GSEA) was performed using GSEA version 2.0 software (Broad Institute; http://www.broadinstitute.org/gsea) with genes ranked by difference of class and statistical significance by 1000 gene set permutations. Gene set permutation was used to enable direct comparisons between shScram and shRNA ZNF521 results (< 7 replicates). Median of probes was used to collapse multiple probe sets to a single value per gene for each sample. Gene sets with a FDR < 0.05 were declared to be statistically significant. The microarray gene expression data have been submitted in NCBI's Gene Expression Omnibus (GEO,http://www.ncbi.nlm.nih.gov/geo/) under accession GSE79110

### Western blot and immunofluorescence staining

Western blot and immunofluorescence were performed using standard procedures. The antibody against ZNF521 was from Novus Biologicals (72009). Gamma-Tubulin (T6557), Actin (A5316), Flag M2 (F3165) antibodies were from Sigma Aldrich. For immunofluorescence analysis, antibody against CDKN1A/p21 (2947S) was from Cell Signaling and antibody against CDKN1B/p27 (610241) was from BD Biosciences. Detail method for the immunofluorescence is described in Supplementary Materials and Methods.

### Cell function analyses

Cell viability, clonogenicity, cell cycle, apoptosis, expression of CD11b and CD14, morphological analysis and cell differentiation induction were performed as detailed in Supplementary Materials and Methods.

### Studies with AML patient-derived xenograft cells

Primary *MLL-AF9*-expressing cells were obtained from BM samples of diagnosed AML pediatric patients stored in the BioBank of the laboratory of Pediatric Hematology of the University Hospital of Padua, (Italy) according to the guidelines of the local ethics committee. Initial AML xenografts were established by tail vein injection with 8 × 10^6^ primary cells suspended in 300 μl of PBS in 6- to 8-week-old NSG mice, which were purchased from Charles River (Wilmington, MA, USA). All animal experiments were performed in accordance with institutional guidelines and established protocols [53]. Engraftment was monitored by weekly blood collections and flow cytometry analysis with antihuman CD45 (BD Biosciences). The engraftment rate was defined by the number of days required for the transplanted human CD45+ cells to reach at least 20% in the peripheral blood. Human leukemic cells from the spleens of engrafted mice were collected and cultivated in RPMI supplemented with 10% Human serum (Euroclone), antibiotics, and cytokines SCF, FLT-3L and TPO (40 ng/ml for each), IL-3 and IL-6 (20 ng/ml for each). (All cytokines were obtained from Inalco, Milan, Italy). For *ex vivo* experiments, two independent biological replicates were performed.

### Data analysis

Data are presented as mean ± SD. Each experiment was performed at least 3 times, except where stated otherwise. The differences were examined using 2-tailed *t* test, Mann–Whitney *U-test* or kruskal-Wallis one-way analysis of variance followed Dunn's test as appropriate (GraphPad Prism; GraphPad). Results were considered significant at *P* < 0.05.

## SUPPLEMENTARY MATERIALS FIGURES AND TABLES




