# Supplementary material for: ZNF521 sustains the differentiation block in MLL-rearranged acute myeloid leukemia
Source: Oncotarget. 2017 Feb 16;8(16):26129–41. doi: 10.18632/oncotarget.15387 (PMC5432245; doi:10.18632/oncotarget.15387)
Supplement: Supplementary file 1 [file oncotarget-08-26129-s001.pdf]

ZNF521 sustains the differentiation block in MLL-rearranged acute myeloid leukemia

Supplementary Materials

MATERIALS AND METHODS

ZNF521 promoter isolation and plasmid constructs

Wild-type *ZNF521* promoter from −4493 to + 65 relative to the transcription start site (TSS) was divided in four genome fragments [P1 (−3810 to 4993), P2 (−2560 to 3970), P3 (−1060 to 2729) and P4, +62 to −1260)] and each one was isolated from a genomic DNA obtained from a pool of buffy coat by PCR amplification using primer pairs containing specific restriction sites. All PCR products were purified, sequenced and cloned in TOPO TA cloning kit (Invitrogen). P1 and P3 plasmids were *Xho*I/*Hind*III-digested and the fragments were recloned into *Xho*I/*Hind*III sites downstream of the luciferase gene of the pGL42.8 luciferase vector (Promega) to generate *ZNF521P1-luc* and *ZNF521P3-luc* constructs, respectively. Similarly, P2 and P4 plasmids were *Nhe*I/*Xho*I-digested and the fragments inserted into *Nhe*I/*Xho*I sites of pGL42.8 vector to generate *ZNF521P2-luc* and *ZNF521P4-luc* constructs, respectively. The genome fragment P3 (−1060 to 2729) was subdivided in three smaller parts [P3.1 (−2101 to −2729), P3.2 (−1533 to −2143) and P3.3 (−1060 to −1610)] and each part was PCR amplified from genomic DNA using primers containing specific restriction sites and cloned in TOPO TA vector. P3.1 and P3.2 plasmids were *Xho*I/*Hind*III-digested and the fragments were recloned into *Xho*I/*Hind*III sites of pGL42.8 vector to generate *ZNF521P3.1-luc* and *ZNF521P3.2-luc* constructs. P3.3 plasmid was *Nhe*I/*Xho*I-digested and the fragment recloned into *Nhe*I/*Xho*I-digested pGL42.8 to generate *ZNF521P3.3* construct. (Primers used for genomic DNA amplification are listed in Supplementary Table 2).

Immunofluorescence staining

For immunofluorescence analysis, 5 × 10^4 FACS-sorted cells were harvested 7 days after transduction and cytocentrifuged onto slides at 500 rpm for 5 minutes (Cytospin 4 cytocentrifuge, Thermo Scientific). Cells were fixed in 4% formaldehyde for 15 minutes, permeabilized with PBS containing 0.1% Triton X-100 (Sigma) for 10 minutes, and blocked with 5% BSA in PBS for 30 minutes at room temperature, followed by incubation with primary antibodies in 1% BSA-PBS overnight at 4°C. The following day, slides were washed with PBS and incubated with the secondary antibody Alexa Fluor 594 Goat Anti-mouse IgG (H+L) (1:2000, Life Technologies) for 1 hour at room temperature. Nuclei were stained with 4′,6-diamidino-2-phenylindole (DAPI, 1:10000; Sigma) for 13 minutes. A minimum of 50 cells/sample was scored in three different fields in at least three independent cytospin preparations. Cells were visualized and counted by a confocal microscope (Vico, Eclipse Ti80, Nikon) equipped with a digital camera. Images were captured with ImageProPlus software (Media Cybernetics).

Cell viability and clonogenic assay

Viability assay was performed using the colormetric diphenyltetrazolium bromide (MTT) Cell Proliferation Kit I (Sigma) and measured via Victor 3 Microplate reader (PerkinElmer). Briefly, FACS-sorted GFP positive cells (5 × 10^4) from cell lines and/or *ex vivo* primary patient-derived cells were collected 4 days after transduction and seeded (in triplicates) in 96-well culture plate. At a designated time point, cell viability was measured by adding 10 ml of MTT solution (5 mg/ml) to each well. After 3 hours of incubation, the absorbance (OD) of each well was measured at 570 nm using a microplate reader (Victor 3; PerkinElmer). Cell proliferation was calculated by the following formula: (mean OD ZNF521-shRNA wells)/(mean OD shScram-control wells) × 100%. For the clonogenic assay, 2 × 10^3 cells at day 4 of transduction were FACS-sorted and then resuspended and seeded in methylcellulose medium (MethoCult H4534; Stem Cell Technologies). After 14 days, colonies were counted under 20× magnification with a stereoscope after exposure to MTT (Sigma) for 3 hours at 37°C.

Cell cycle, apoptosis assay

Cell-cycle analysis was performed by flow cytometric analysis of propidium iodide-stained cells at 7 days after shRNA transduction. Briefly, 2.5 × 10^5 cells were washed in PBS and then fixed in 70% ethanol and put at −20°C overnight. The samples were rehydrated in cold PBS, treated with lysis buffer containing RNase and 0.1% Triton X-100 (Sigma) and stained with propidium iodide (PI). Cell cycle distribution was measured on gated GFP-positive cells. Analyses were performed using the Cytomics FC500 flow cytometer (Beckman

Coulter, Brea, CA) and MultiCycle Cell Cycle Analysis Software (Phoenix Flow Systems, San Diego, CA). A minimum total of 10.000 gated events were collected for each sample. Cell death was measured at day 4 and day 7 after transduction using AnnexinV-Fluos staining kit (Invitrogen) and analyzed by flow cytometry. Briefly, 2.5×10^5 cells were resuspended in 100 ml of 1× annexinV-binding buffer and incubated with 1 ml of allophycocyanin-conjugated annexinV (Invitrogen) and 1 ml of DAPI (1:10000, Sigma) for 15 minutes at room temperature. The apoptotic cells (AnnexinV$^+$/DAPI−) were determined on gated GFP-positive cells as described above.

Expression of CD11b and CD14, morphological analysis and cell differentiation induction

PE-conjugated anti-CD11b (BD Biosciences) and PE-Cyanin7-coniugated anti-CD14 (Beckman Coulter) were used to analyze myeloid differentiation. Briefly, on day 7, 2×10^5 transduced cells were harvested, washed and labeled with conjugated antibodies in PBS for 15 minutes in the dark. Then, cells were washed and analyzed by flow cytometry of gated GFP-expressing cells as previously reported. The expression of the cell surface markers was analyzed with a Cytomics FC500 flow cytometer ((Beckman Coulter, Brea, CA). Cell morphology was determined on cytospin preparations. Four days after transduction, GFP positive cells were FACS-sorted and placed in culture. Three days later (7 days post-transduction), 0.5×10^5 cells were harvested and washed in PBS buffer and spun onto slides for 5 minutes at 500 rpm using a Shandon CytoSpin4 cytocentrifuge and the slides were stained with a Wright-Giemsa stain. Images were taken at 40× magnification using a Nikon microscope (Vico, Eclipse Ti80, Nikon) and acquired with ImageProPlus software (Media Cybernetics). For the differentiation induction with ATRA (1 μM; Sigma) or Securinine (15 μM; Sigma), after 3 days of treatment cells were collected and analyzed for cell morphology, level of ZNF521 transcripts and protein expression as previously described.

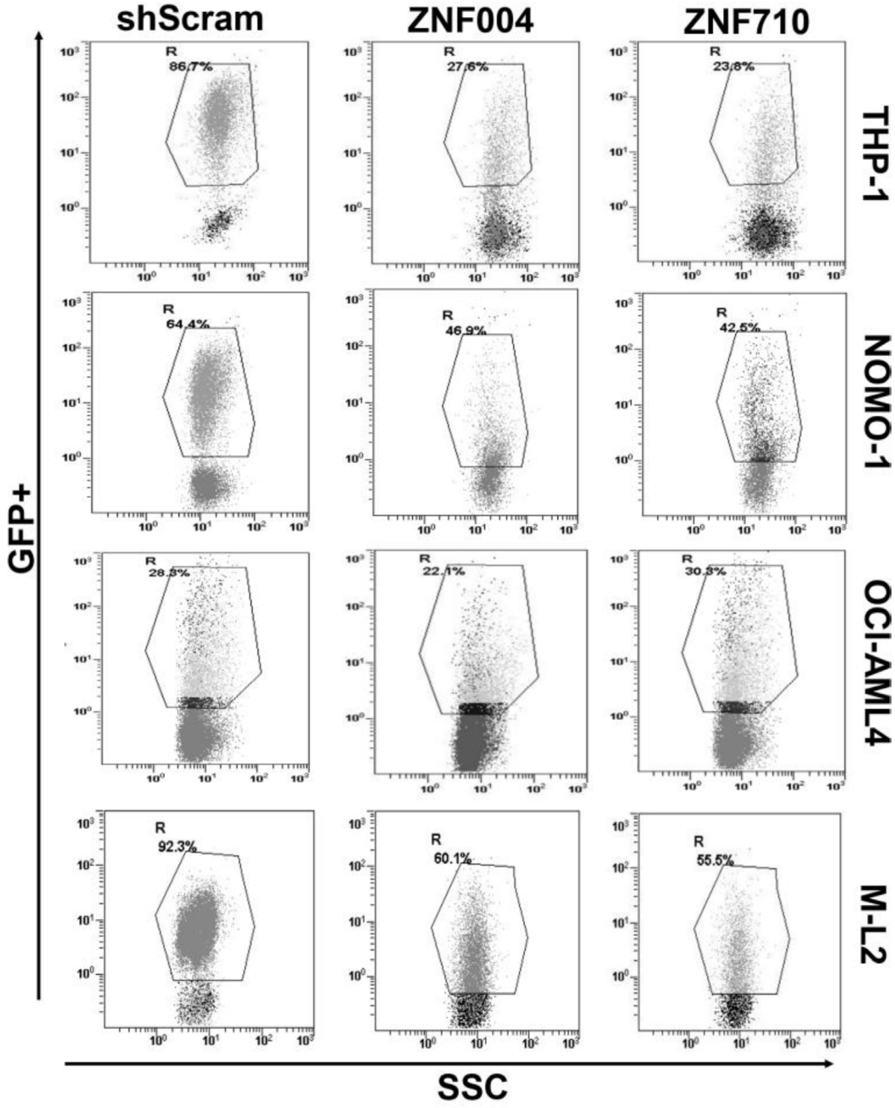

Supplementary Figure 1: Flow cytometry analysis and gating strategy of shRNA transduced human *MLL*-rearranged AML cell lines. Transduced cell lines were GFP-sorted at day 4 and analyzed or maintained in culture for further evaluation. Otherwise transduced cells were analyzed after being gated for the GFP+ cells. Representative flow cytometry dot plots of gated GFP+ cells after transduction with GFP lentiviral expressing shRNA against *ZNF521* (ZNF004 and ZNF710) or expressing non-targeting shRNA sequence (shScram) are presented. The selected area indicates the sorting gates and includes the percentage of cells in each sorting gate. Gates were set to collect GFP high and GFP low-expressing cells. SSC, side scatter.

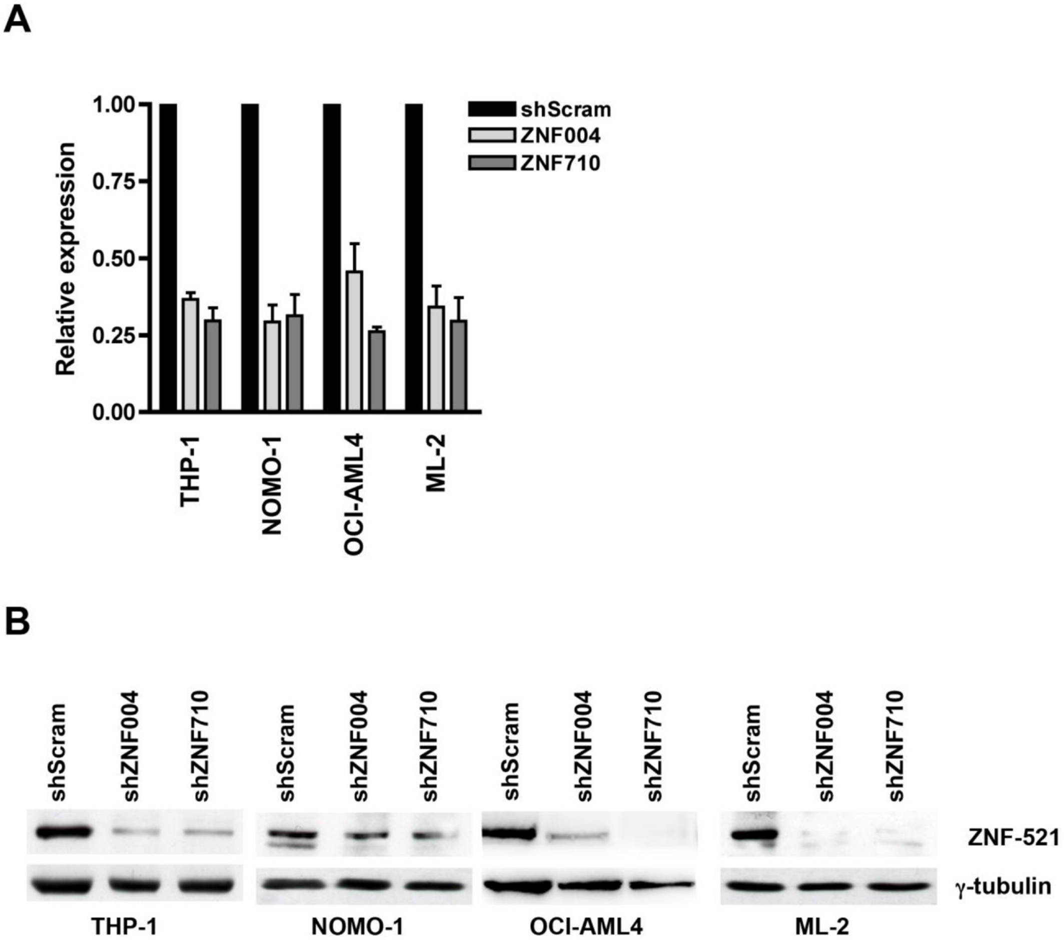

Supplementary Figure 2: shRNA-mediated knockdown of *ZNF521* in *MLL*-rearranged AML cell lines. (**A**) *ZNF521* mRNA levels evaluated by qRT-PCR on GFP+ sorted cells after 4 days of transduction with shScram and *ZNF521* shRNAs (ZNF004 and ZNF710). The results are relative to shScram-transduced cells, normalized to *GAPDH* and analyzed by $2^{-\Delta\Delta Ct}$ method. Data are represented as mean ± SD of three independent experiments. (**B**) Western blot analysis for ZNF521 of cells used in (A). γ-tubulin was used as loading control.

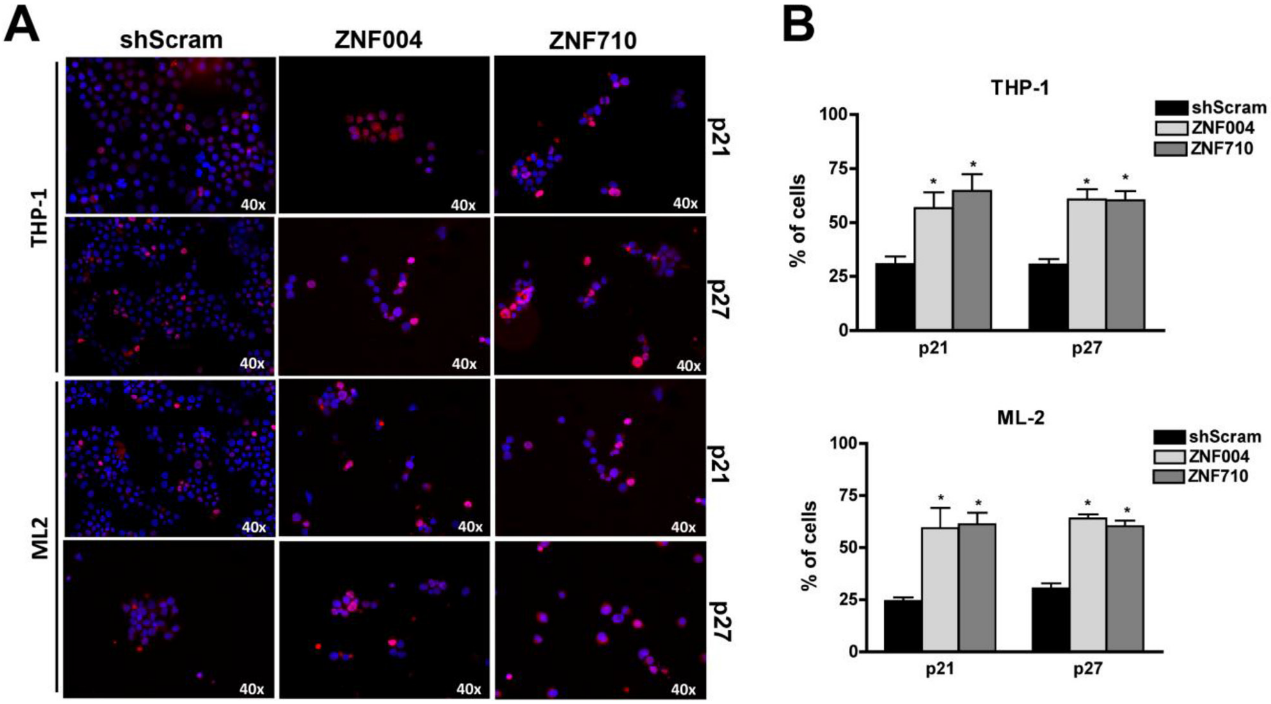

Supplementary Figure 3: *ZNF521* depletion increases the expression signal of p21 and p27. (**A**) Representative confocal imaging on cytospin preparations of THP-1 and ML-2 cell lines analyzed for p21 and p27 expression by immunofluorescence assay. Transduced cells with shScram or ZNF521 shRNAs (ZNF004 or ZNF710) at day 7 were stained with anti-p21 and anti-p27 antibodies and Alexa Fluor 594-conjugated goat anti-mouse IgG (red). Corresponding nuclei were counterstained with DAPI (blue). Original magnification, × 40. Images were collected by confocal microscope (Vico, Eclipse Ti80, Nikon) and processed with ImageProPlus software (Media Cybernetics). Results are representative of at least three independent experiments for each cell line. (**B**) Percentage of p21 and p27 positive cells was quantified respect to total nuclei (see supplemental method). Data are mean ± SD of three cytospin preparations for each cell line of three independent experiments. $*P < 0.05$, *t*-test

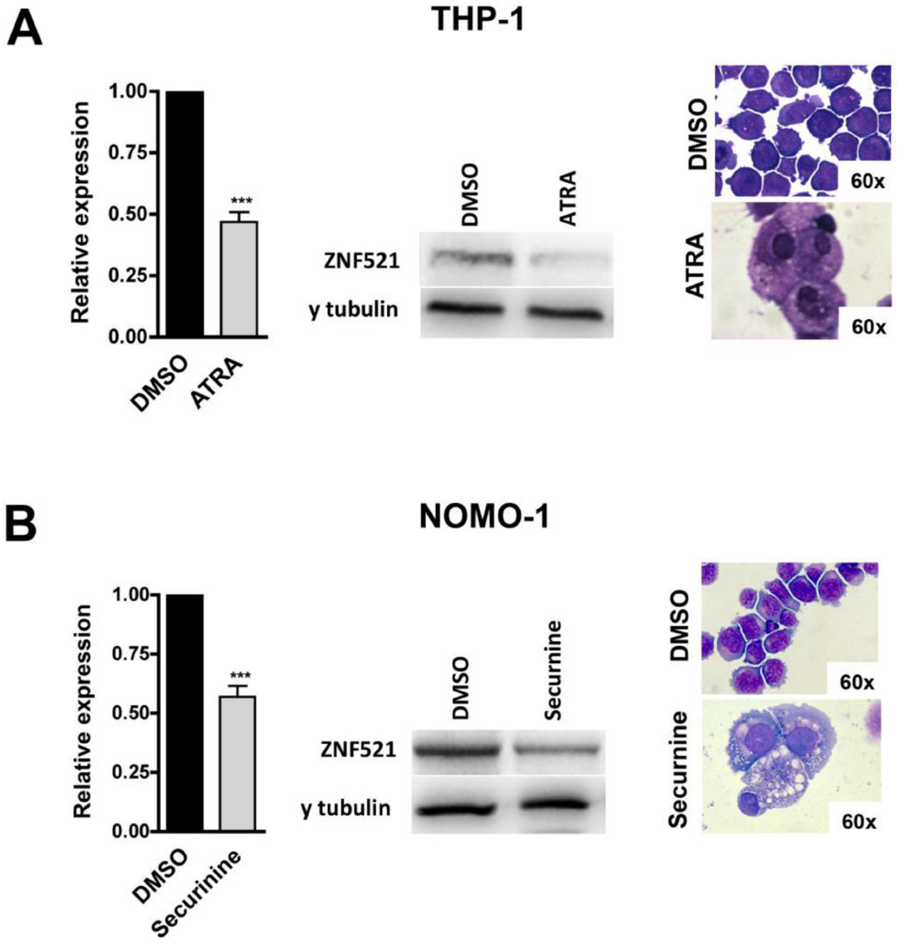

Supplementary Figure 4: Effect of differentiation-induced agents on *ZNF521* expression in human *MLL*-rearranged cell lines. (**A**) qRT-PCR (left panel) and Western blot (middle panel) analyses of ZNF521 in THP-1 cell line after treatment with 1 μM ATRA for 72 hours compared with vehicle control (0.1% DMSO). For qRT-PCR data the *ZNF521* mRNA expression is shown relative to vehicle control normalized to *GAPDH* and analyzed by $2^{-\Delta\Delta Ct}$ method. Data are represented as mean ± SD of three independent experiments. ***$P < 0.001$, *t*-test. Representative Wright-Giemsa-stained cytospin preparations of cells treated with 1 μM ATRA for 72 hours (right panel). Original magnification, × 60. (**B**) Same experiments as in (A) were performed in NOMO-1 cell line after 72 hours of treatment with 15 μM Securinine. qRT-PCR (left panel), Western blot (middle panel) analyses of ZNF521 expression and representative Wright-Giemsa-stained cytospin preparations of NOMO-1 cell lines after 72 hours of treatment (right panel) are shown.

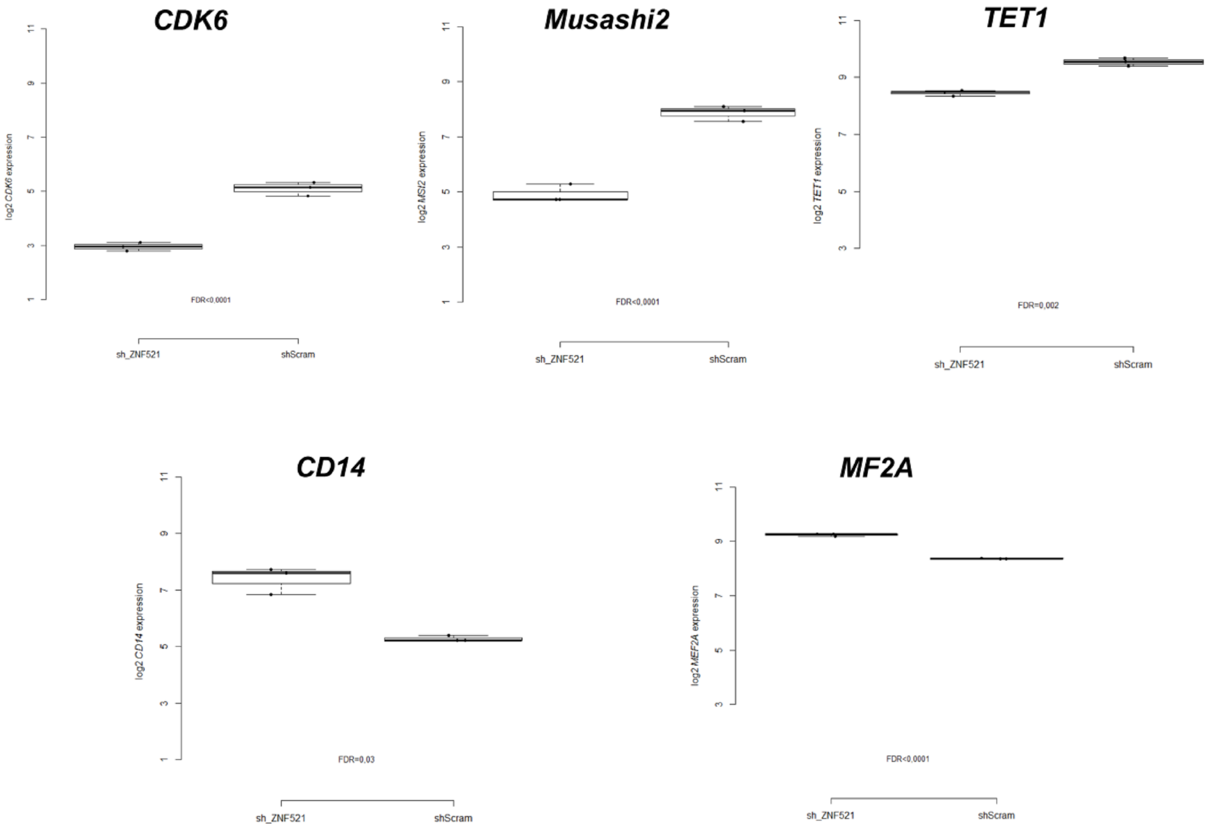

Supplementary Figure 5: Box-plots showing the expression levels of CDK6, *MSI2*, TET1, CD44 and MEF2A. These genes were selected from microarray data of transduced THP-1 cells with ZNF521 shRNA (ZNF004) or shScram. FDR, false discovery rate.

Supplementary Table 1: Genotype features of the 50 childhood AML patients analyzed for *ZNF521* expression by qRT-PCR analysis

Non-*MLL*-rearranged (*n* = 34)		*MLL*-rearranged (*n* = 16)	
normal karyotype	13 (38%)	t(10;11)(*MLL-AF10*)	5 (31%)
t(15;17)(*PML-RARα*)	6 (18%)	t(9;11)(*MLL-AF9*)	5 (31%)
inv16(*CBFB-MYH11*)	3 (9%)	t(6;11)(*MLL-AF6*)	3 (19%)
t(8;21)(*AML1-ETO*)	8 (23%)	11q23 others *	3 (19%)
FLT3-ITD +	4 (12%)		

*t(11;19)(*MLL-ENL*) *n* = 1; t(11;19)(*MLL-ELL*) *n* = 1; t(X;11)(*MLL-SEPTIN6*) *n* = 1.

Supplementary Table 2: Sequence of the primers used in this study

Primers for qRT-PCR

Gene name	Forward 5′ → 3′	Reverse 5′ → 3′
GAPDH	AGGGCTGCTTTTAACTCTGGT	CCCCACTTGATTTTGGAGGGA
ZNF521	ACTGAAGTTTGGCAGGAGAG	TGGGATATTCAGGTTCATGTT
PU.1	AGAAGACCTGGTGCCCTA	CCAGTAATGGTCGTCATGGC
C/EBPα	AACATCGCGGTGCGCAAGAG	TTCGCGCTCAGCTGTTCCA
HOXA9	AAAACAATGCTGAGAATGAGAGC	TATAGGGGCACCGCTTTTT

Primers for ZNF521 promoter^

Gene name	Forward 5′ → 3′	Reverse 5′ → 3′
ZNF521P1	AGCTGCTAGCACATTAAACTATACCAAAGAAATCCA	AGCTAAGCTTAAAACTATACTCAGTTCCCAGTTCC
ZNF521P2	AGCTGCTAGCTCCATGTGACGTTCTTAAATGC	AGCTGCTAGCTTAGCCACTGCAGAAAGGTAAA
ZNF521P3	AGCTGCTAGCTGACGTTCTCATTGTAGCTGGT	AGCTAAGCTTATGAAGCCAAAGCCATCATC
ZNF521P4	AGCTGCTAGCTCCAGGCAGTTTACAGGTTAGA	AGCTGCTAGCCTGTACGTAATCACTGAGGAAATCAT
ZNF521P3.1	AGCTGCTAGCTGACGTTCTCATTGTAGCTGGT	AGCTAAGCTTATGAAGCCAAAGCCATCATC
ZNF521P3.2	AGCTGCTAGCAAGTTGCTGCATTCTGCTCA	AGCTAAGCTTTTTCCTTTCGTGTGGTAGCC
ZNF521P3.3	AGCTGCTAGCTATCACACATAACTTGGGACCAC	AGCTCTCGAGGTGGAAATTAAGAGATTCAGAATACG

Primers for ChIP analysis

Gene name	Forward 5′ → 3′	Reverse 5′ → 3′
HOXA9	AATGCGATTTGGCTGCTTTTTTATGGC	TCAAATCTGGCCTTGCCTCTG
ZNF521p3.3	ACACATAACTTGGGACCACAC	GTCTAACCTGTAAACTGCCTGGA
**shRNA sequences in Mission shRNA pLKO.1-puro-CMV-TurboGFP**		
shZNF521_9004	GTACCGGATCACTTGAAGATCCACTTAACTCGAGTTAAGTGGATCTTCAAGTGATTTTTTTG	
shZNF521_9710	CCGGACAAGTTGCAGCAGCATATTTCTCGAGAAATATGCTGCTGCAACTTGTTTTTG	
shScram	GGACAAGTTGCAGCAGCATATTTCTCGAGAAATATGCTGCTGCAACTTGTTTTTT	

^The restriction enzyme sites are underlined.

Supplementary Table 3: Clinical features of patients for xenotransplantation into NOD/SCID mice

ID	WBC, × 10^9/L	FAB	% BM blasts at diagnosis	Disease status at biopsy	karyotype
1426	37.5	NA	90	Diagnosis	46,XY,t(9;11)(p22;q23)
726*	NA	M5	80	Diagnosis	46,XY,t(9;11)(p22;q23)
1315*	85.2	NA	80	Secondary	46,XY,7p,t(9;11)(p22;q23)
1368	222.4	NA	80	Diagnosis	46,XY,t(9;11)(p22;q23)

*Sample-patient successfully engrafted; NA not available.

Supplementary Table 4: THP-1 microarray (genes deregulated with > 1.5-fold change, FDR < 0.05).

See Supplementary_Table_4
