# Supplementary material for: ZNF521 sustains the differentiation block in MLL-rearranged acute myeloid leukemia
Source: Oncotarget. 2017 Feb 16;8(16):26129–41. doi: 10.18632/oncotarget.15387 (PMC5432245; doi:10.18632/oncotarget.15387)
Supplement: Supplementary file 2 [file oncotarget-08-26129-s002.docx]

**Table S4**

**THP-1 microarray (genes deregulated with >1.5-fold change, FDR<0.05)**

| **Probe set** | **Gene Symbol** | **IFDR** | **Means_shZNF004** | **Means_shScram** |
| --- | --- | --- | --- | --- |
| 1555340_x_at | *RAP1A* | 7.29E-14 | 3.272817153 | 11.69366146 |
| 1555339_at | *RAP1A* | 7.29E-14 | 3.331697417 | 11.72192487 |
| 203032_s_at | *FH* | 7.29E-14 | 2.593654642 | 7.004073844 |
| 230659_at | *NA* | 7.29E-14 | 3.364707971 | 9.071419646 |
| 209811_at | *CASP2* | 7.29E-14 | 3.498066564 | 6.977897606 |
| 1555830_s_at | *ESYT2* | 7.29E-14 | 3.157621165 | 6.233288774 |
| 228854_at | *NA* | 7.29E-14 | 4.507943084 | 8.700411129 |
| 1554451_s_at | *DNAJC14* | 7.29E-14 | 3.443528107 | 6.599509088 |
| 222611_s_at | *PSPC1* | 7.29E-14 | 3.892862189 | 6.940868082 |
| 216125_s_at | *RANBP9* | 7.29E-14 | 4.214434937 | 6.786602432 |
| 227762_at | *NA* | 7.29E-14 | 4.760415465 | 7.656360326 |
| 242900_at | *NA* | 7.29E-14 | 3.972371741 | 6.289884015 |
| 210465_s_at | *SNAPC3* | 7.29E-14 | 5.260781091 | 8.320937398 |
| 209052_s_at | *WHSC1* | 7.29E-14 | 4.011707509 | 6.321660403 |
| 224577_at | *ERGIC1* | 7.29E-14 | 5.026229524 | 7.551517562 |
| 219209_at | *IFIH1* | 7.29E-14 | 9.661284664 | 6.360666226 |
| 227834_at | *TXLNB* | 7.29E-14 | 5.734833216 | 3.702313322 |
| 219243_at | *GIMAP4* | 7.29E-14 | 5.377588503 | 3.461148283 |
| 216598_s_at | *CCL2* | 7.29E-14 | 8.428115374 | 5.381391593 |
| 203596_s_at | *IFIT5* | 7.29E-14 | 7.756051699 | 4.938882008 |
| 227265_at | *FGL2* | 7.29E-14 | 6.933841791 | 4.350212792 |
| 210889_s_at | *FCGR2B* | 7.29E-14 | 9.3858295 | 5.882368351 |
| 223551_at | *PKIB* | 7.29E-14 | 9.164302426 | 5.661218939 |
| 204475_at | *MMP1* | 7.29E-14 | 7.330288469 | 4.387222797 |
| 229450_at | *IFIT3* | 7.29E-14 | 11.66175734 | 6.924802655 |
| 206488_s_at | *CD36* | 7.29E-14 | 8.995345742 | 5.33583655 |
| 228766_at | *CD36* | 7.29E-14 | 7.475487386 | 4.405886495 |
| 205686_s_at | *CD86* | 7.29E-14 | 5.675449214 | 3.327806002 |
| 210895_s_at | *CD86* | 7.29E-14 | 7.799936929 | 4.402895353 |
| 202973_x_at | *FAM13A* | 7.29E-14 | 7.555883194 | 4.23511804 |
| 231120_x_at | *PKIB* | 7.29E-14 | 6.821698244 | 3.738890628 |
| 204972_at | *OAS2* | 7.29E-14 | 7.437693218 | 3.962123467 |
| 209555_s_at | *CD36* | 7.29E-14 | 7.849275842 | 4.152806423 |
| 206584_at | *LY96* | 7.29E-14 | 7.396080064 | 3.888310801 |
| 226757_at | *IFIT2* | 7.29E-14 | 10.04575707 | 5.214422189 |
| 206637_at | *P2RY14* | 7.29E-14 | 6.443625151 | 3.174919145 |
| 212956_at | *TBC1D9* | 7.29E-14 | 8.199774221 | 3.989995117 |
| 227609_at | *EPSTI1* | 7.29E-14 | 10.61497537 | 4.856721475 |
| 202086_at | *MX1* | 7.29E-14 | 11.59476291 | 5.268245207 |
| 203153_at | *IFIT1* | 7.29E-14 | 10.51899342 | 4.701311253 |
| 235276_at | *EPSTI1* | 7.29E-14 | 8.531841866 | 3.790497541 |
| 214453_s_at | *IFI44* | 7.29E-14 | 10.18360475 | 4.365661325 |
| 204439_at | *IFI44L* | 7.29E-14 | 9.343279892 | 2.623412862 |
| 208450_at | *LGALS2* | 2.72E-12 | 6.376452482 | 4.13836002 |
| 230520_at | *AIG1* | 3.62E-12 | 5.421414771 | 8.42334648 |
| 225237_s_at | *MSI2* | 3.62E-12 | 4.302027085 | 6.63526392 |
| 209875_s_at | *SPP1* | 3.62E-12 | 8.939636755 | 5.245139693 |
| 239512_at | *SRSF4* | 5.43E-12 | 3.424633107 | 6.122115045 |
| 215099_s_at | *RXRB* | 5.43E-12 | 3.360896669 | 5.182917746 |
| 214022_s_at | *IFITM1* | 1.09E-11 | 9.639456366 | 5.780716705 |
| 227242_s_at | *EBF3* | 1.40E-11 | 3.198777829 | 6.501337123 |
| 209160_at | *AKR1C3* | 1.40E-11 | 6.237174611 | 3.467544531 |
| 209409_at | *GRB10* | 1.52E-11 | 5.53032405 | 8.592046019 |
| 226841_at | *MPEG1* | 1.52E-11 | 5.439137025 | 3.463256631 |
| 206291_at | *NTS* | 8.04E-11 | 5.202834617 | 3.37912861 |
| 235625_at | *VPS41* | 1.12E-10 | 4.019442688 | 6.888053529 |
| 219352_at | *HERC6* | 1.12E-10 | 8.512887407 | 5.127299556 |
| 242625_at | *RSAD2* | 1.12E-10 | 7.759363805 | 3.535304072 |
| 227260_at | *NA* | 1.68E-10 | 3.468106472 | 5.812574528 |
| 229128_s_at | *ANP32E* | 1.92E-10 | 3.065627289 | 8.041154478 |
| 211825_s_at | *FLI1* | 2.56E-10 | 4.505153042 | 7.70808605 |
| 204273_at | *EDNRB* | 1.21E-09 | 4.644985144 | 3.081504207 |
| 220059_at | *STAP1* | 1.21E-09 | 6.948836147 | 3.9587154 |
| 34449_at | *CASP2* | 2.22E-09 | 3.371752104 | 5.343900903 |
| 202869_at | *OAS1* | 2.40E-09 | 8.708784226 | 3.999158316 |
| 209925_at | *OCLN* | 2.53E-09 | 3.66215453 | 5.709879327 |
| 213562_s_at | *SQLE* | 2.70E-09 | 3.41534405 | 6.727026189 |
| 219895_at | *TMEM255A* | 3.97E-09 | 5.912972966 | 2.952282219 |
| 212681_at | *EPB41L3* | 6.35E-09 | 6.875338979 | 4.226037005 |
| 1552658_a_at | *NAV3* | 2.21E-08 | 4.719195295 | 7.76328231 |
| 210139_s_at | *PMP22* | 2.21E-08 | 6.202295096 | 3.699815143 |
| 229167_at | *PURA* | 4.17E-08 | 4.433892969 | 6.889873925 |
| 217403_s_at | *ZNF227* | 5.67E-08 | 3.767873968 | 6.526712195 |
| 211559_s_at | *CCNG2* | 6.59E-08 | 4.422448321 | 8.428323945 |
| 219196_at | *SCG3* | 6.59E-08 | 5.429712116 | 2.812053741 |
| 213872_at | *NA* | 1.09E-07 | 3.010296172 | 7.791453316 |
| 201693_s_at | *EGR1* | 1.09E-07 | 4.539918709 | 7.633098497 |
| 210875_s_at | *ZEB1* | 1.09E-07 | 2.896438208 | 4.421607261 |
| 222614_at | *RWDD2B* | 1.98E-07 | 3.450423478 | 5.86072941 |
| 1553117_a_at | *STK38* | 2.10E-07 | 3.665817621 | 6.939115251 |
| 244774_at | *PHACTR2* | 2.10E-07 | 3.853094162 | 6.851288289 |
| 233292_s_at | *NA* | 3.41E-07 | 3.184355172 | 4.827088859 |
| 214539_at | *SERPINB10* | 4.15E-07 | 4.10912414 | 7.005074609 |
| 224046_s_at | *PDE7A* | 6.57E-07 | 3.65311398 | 6.146994073 |
| 227803_at | *ENPP5* | 6.57E-07 | 4.717081416 | 3.098961165 |
| 216917_s_at | *SYCP1* | 6.57E-07 | 5.727581378 | 3.442402712 |
| 209723_at | *SERPINB9* | 6.57E-07 | 6.525317888 | 3.87000973 |
| 206336_at | *CXCL6* | 6.57E-07 | 5.61551641 | 3.22831843 |
| 1554614_a_at | *PTBP2* | 9.72E-07 | 2.851477818 | 6.01832904 |
| 211450_s_at | *MSH6* | 9.72E-07 | 3.945522181 | 7.983327736 |
| 1558028_x_at | *LINC00657* | 9.72E-07 | 3.881579348 | 6.677504769 |
| 205408_at | *MLLT10* | 9.72E-07 | 4.396444854 | 7.251866597 |
| 243835_at | *ZDHHC21* | 1.29E-06 | 3.131394065 | 4.933095838 |
| 212009_s_at | *STIP1* | 1.29E-06 | 4.231623821 | 6.593862637 |
| 235287_at | *CDK6* | 2.42E-06 | 2.962197505 | 5.106066397 |
| 226134_s_at | *MSI2* | 2.42E-06 | 4.910426996 | 7.85923352 |
| 242691_at | *NA* | 3.29E-06 | 3.255871284 | 5.303849989 |
| 225864_at | *FAM84B* | 3.29E-06 | 3.477901748 | 5.450077376 |
| 223220_s_at | *PARP9* | 3.29E-06 | 10.1340089 | 6.754157491 |
| 205139_s_at | *UST* | 3.29E-06 | 6.260118791 | 4.052990312 |
| 205997_at | *ADAM28* | 3.29E-06 | 6.747742756 | 4.12818658 |
| 207723_s_at | *KLRC3* | 3.29E-06 | 6.848721055 | 3.928225706 |
| 222863_at | *ZBTB10* | 5.30E-06 | 3.003991336 | 4.715064116 |
| 228834_at | *TOB1* | 6.78E-06 | 3.10919356 | 6.508586179 |
| 237346_at | *TGDS* | 6.78E-06 | 2.659434222 | 4.423719391 |
| 239245_at | *NA* | 7.78E-06 | 2.830149135 | 4.556059038 |
| 234023_s_at | *CENPJ* | 8.03E-06 | 2.838765479 | 5.393589327 |
| 213470_s_at | *HNRNPH1* | 8.03E-06 | 4.388092403 | 7.884930732 |
| 228746_s_at | *NA* | 1.37E-05 | 3.420014404 | 7.3567208 |
| 222719_s_at | *PDGFC* | 1.37E-05 | 3.132188837 | 5.747502104 |
| 226302_at | *ATP8B1* | 1.37E-05 | 4.729303393 | 2.953088663 |
| 205992_s_at | *IL15* | 1.37E-05 | 5.108587768 | 3.02186665 |
| 202411_at | *IFI27* | 1.37E-05 | 10.32098553 | 4.155674395 |
| 205660_at | *OASL* | 2.00E-05 | 8.816232559 | 5.043755348 |
| 201601_x_at | *NA* | 3.82E-05 | 9.514157211 | 6.172838679 |
| 1553105_s_at | *DSG2* | 8.06E-05 | 3.716787789 | 7.380013058 |
| 1555745_a_at | *LYZ* | 8.65E-05 | 3.657795152 | 8.966469996 |
| 204156_at | *SIK3* | 0.000191614 | 3.014616564 | 5.596974796 |
| 205883_at | *ZBTB16* | 0.000191614 | 3.9323087 | 6.382959729 |
| 220773_s_at | *GPHN* | 0.000191614 | 3.965163502 | 6.331618458 |
| 231955_s_at | *HIBADH* | 0.000191614 | 4.994812536 | 7.532474333 |
| 205003_at | *DOCK4* | 0.000191614 | 6.547858789 | 4.336486424 |
| 231504_at | *CCDC148* | 0.000191614 | 5.515141165 | 3.546226457 |
| 228607_at | *OAS2* | 0.000191614 | 6.369024331 | 3.246325238 |
| 208055_s_at | *HERC4* | 0.000210734 | 3.689389873 | 6.041702168 |
| 209535_s_at | *NA* | 0.000283867 | 3.889135907 | 6.781673843 |
| 1555154_a_at | *QKI* | 0.000283867 | 4.751795874 | 7.783719526 |
| 215991_s_at | *EMC1* | 0.000283867 | 2.249362269 | 3.683270007 |
| 231274_s_at | *NA* | 0.000283867 | 3.425324062 | 5.435462999 |
| 204698_at | *ISG20* | 0.000283867 | 8.165708071 | 5.327347603 |
| 1554411_at | *CTNNB1* | 0.000376125 | 3.572852599 | 6.635933907 |
| 219599_at | *EIF4B* | 0.000376125 | 4.228875606 | 7.030587927 |
| 209055_s_at | *CDC5L* | 0.000376125 | 5.005916825 | 8.112529596 |
| 220241_at | *TMCO3* | 0.000376125 | 3.662690418 | 5.589867462 |
| 229787_s_at | *OGT* | 0.000598768 | 2.377767023 | 4.198765755 |
| 221423_s_at | *YIPF5* | 0.000598768 | 4.764427984 | 7.983886289 |
| 218031_s_at | *FOXN3* | 0.000598768 | 5.358068359 | 8.479315641 |
| 227978_s_at | *ZADH2* | 0.000598768 | 4.560782669 | 6.978366066 |
| 239827_at | *RGCC* | 0.000737413 | 2.922811434 | 4.531183441 |
| 205552_s_at | *OAS1* | 0.000994295 | 8.52556634 | 4.199854718 |
| 239131_at | *NA* | 0.001104595 | 2.869233639 | 4.766296652 |
| 241699_at | *NA* | 0.001104595 | 2.524064046 | 3.903572868 |
| 230265_at | *NA* | 0.001251697 | 3.971358386 | 7.268866264 |
| 206420_at | *IGSF6* | 0.001251697 | 5.105353547 | 3.145928394 |
| 213797_at | *RSAD2* | 0.001251697 | 7.200654765 | 4.091812827 |
| 233878_s_at | *XRN2* | 0.001283386 | 4.250416536 | 8.047670115 |
| 208047_s_at | *NAB1* | 0.001289249 | 2.984541409 | 4.570452219 |
| 201295_s_at | *WSB1* | 0.001327936 | 3.992678087 | 7.276172187 |
| 216015_s_at | *NLRP3* | 0.001658291 | 3.957640333 | 6.742987942 |
| 205996_s_at | *AK2* | 0.001955832 | 6.117002334 | 9.595158229 |
| 1552275_s_at | *PXK* | 0.002059935 | 4.965871756 | 8.855475104 |
| 1569362_at | *ALCAM* | 0.002059935 | 2.854703141 | 4.974867187 |
| 204426_at | *TMED2* | 0.002059935 | 4.787864769 | 8.339465929 |
| 1555996_s_at | *NA* | 0.002059935 | 3.442752363 | 5.602525425 |
| 215109_at | *RC3H1* | 0.002059935 | 3.362155971 | 5.244661583 |
| 235306_at | *GIMAP8* | 0.002059935 | 4.411824265 | 2.656029179 |
| 206544_x_at | *SMARCA2* | 0.00258171 | 4.255382491 | 7.791534959 |
| 1555526_a_at | *6-Sep* | 0.00258171 | 3.226981218 | 5.427391893 |
| 243751_at | *CHD2* | 0.002932455 | 2.862859638 | 4.892477052 |
| 224455_s_at | *ADPGK* | 0.002932455 | 4.545940524 | 7.377039775 |
| 227404_s_at | *EGR1* | 0.002932455 | 6.017690069 | 9.554573831 |
| 242277_at | *NA* | 0.004064267 | 2.948818284 | 4.971712234 |
| 1554433_a_at | *ZNF146* | 0.004064267 | 4.586934708 | 7.705354771 |
| 225742_at | *MDM4* | 0.004064267 | 3.634920337 | 5.721579115 |
| 201075_s_at | *SMARCC1* | 0.004064267 | 5.677039327 | 8.919860071 |
| 220220_at | *NA* | 0.004064267 | 2.721459192 | 4.259068431 |
| 205746_s_at | *ADAM17* | 0.004064267 | 3.986940892 | 6.111574752 |
| 232412_at | *FBXL20* | 0.004064267 | 3.011811652 | 4.544990573 |
| 221039_s_at | *ASAP1* | 0.004609795 | 5.191490691 | 8.244705505 |
| 224582_s_at | *NUCKS1* | 0.004609795 | 4.953985417 | 7.709158094 |
| 239511_s_at | *SRSF4* | 0.004609795 | 2.671862785 | 4.037735602 |
| 205321_at | *EIF2S3* | 0.009657355 | 4.505151046 | 9.214797982 |
| 211089_s_at | *NEK3* | 0.009657355 | 3.153835382 | 5.417381302 |
| 205123_s_at | *NA* | 0.009657355 | 3.148629261 | 5.292748903 |
| 202269_x_at | *GBP1* | 0.009657355 | 5.830633782 | 3.51352209 |
| 240771_at | *C1orf101* | 0.009657355 | 5.03064457 | 2.797264367 |
| 203331_s_at | *INPP5D* | 0.009973264 | 4.808554468 | 7.500169639 |
| 1570552_at | *NA* | 0.009973264 | 2.861867372 | 4.310545092 |
| 239979_at | *NA* | 0.009973264 | 6.28946785 | 4.050437594 |
| 238511_at | *UBL7-AS1* | 0.014726225 | 3.881133701 | 6.361624354 |
| 206785_s_at | *NA* | 0.014726225 | 4.160456492 | 2.549445938 |
| 209754_s_at | *TMPO* | 0.017790649 | 3.93715112 | 6.449468774 |
| 238846_at | *TNFRSF11A* | 0.017790649 | 4.856236144 | 3.122126895 |
| 220735_s_at | *SENP7* | 0.02170125 | 2.719227978 | 4.817388541 |
| 214908_s_at | *TRRAP* | 0.02170125 | 3.580343454 | 5.589555234 |
| 233303_at | *NA* | 0.023432571 | 3.961680025 | 7.556922502 |
| 231918_s_at | *GFM2* | 0.023432571 | 4.303872868 | 7.352626308 |
| 210786_s_at | *FLI1* | 0.03509244 | 5.492847526 | 9.107798461 |
| 216593_s_at | *PIGC* | 0.03509244 | 4.955690919 | 7.569466728 |
| 229540_at | *RBPJ* | 0.03509244 | 3.648570198 | 5.510346371 |
| 201971_s_at | *ATP6V1A* | 0.041216993 | 3.606919921 | 6.108703746 |
| 227364_at | *NA* | 0.041216993 | 6.426258024 | 10.80594044 |
| 222922_at | *KCNE3* | 0.041216993 | 2.993656568 | 4.937417089 |
| 234977_at | *ZADH2* | 0.041216993 | 3.844053383 | 5.942264376 |
| 227299_at | *CCNI* | 0.041216993 | 4.038234393 | 6.224050281 |
| 207782_s_at | *PSEN1* | 0.041216993 | 4.654952249 | 7.164965461 |
| 1553685_s_at | *SP1* | 0.041216993 | 3.317092537 | 5.001010317 |

­­­
